# Elucidating nematode diversity and prevalence in moose across a wide latitudinal gradient using DNA metabarcoding^☆^^☆☆^

**DOI:** 10.1016/j.ijppaw.2024.100962

**Published:** 2024-07-05

**Authors:** Jason L. Anders, Marie Davey, Bram Van Moorter, Frode Fossøy, Sanne Boessenkool, Erling J. Solberg, Erling L. Meisingset, Atle Mysterud, Christer M. Rolandsen

**Affiliations:** a Centre for Ecological and Evolutionary Synthesis (CEES), Department of Biosciences, University of Oslo, P.O. Box 1066 Blindern, NO-0316 Oslo, Norway; b Norwegian Institute for Nature Research (NINA), P. O. Box 5685 Sluppen, NO-7485 Trondheim, Norway; c Norwegian Institute for Nature Research (NINA), Sognsveien 68, 0855 Oslo, Norway; d Department of Forest and Forest Resources, Norwegian Institute of Bioeconomy Research, Tingvoll gard, NO-6630, Tingvoll, Norway

**Keywords:** Alces alces, Migration, Habitat use, Moose, Nematode diversity

## Abstract

Parasitic nematodes are ubiquitous and can negatively impact their host by reducing fecundity or increasing mortality, yet the driver of variation in the parasite community across a wildlife host's geographic distribution remains elusive for most species. Based on an extensive collection of fecal samples (n = 264) from GPS marked moose (*Alces alces*), we used DNA metabarcoding to characterize the individual (sex, age class) and seasonal parasitic nematode community in relation to habitat use and migration behavior in five populations distributed across a wide latitudinal gradient (59.6°N to 70.5°N) in Norway. We detected 21 distinct nematode taxa with the six most common being *Ostertagia* spp., *Nematodirella* spp., *Trichostongylus* spp., *T*. *axei*, *Elaphostrongylus alces,* and an unclassified Strongylida. There was higher prevalence of livestock parasites in areas with larger sheep populations indicating a higher risk of spillover events. The individual level nematode richness was mostly consistent across study areas, while the number and type of nematode taxa detected at each study area varied considerably but did not follow a latitudinal gradient. While migration distance affected nematode beta-diversity across all sites, it had a positive effect on richness at only two of the five study areas suggesting population specific effects. Unexpectedly, nematode richness was higher in winter than summer when very few nematodes were detected. Here we provide the first extensive description of the parasitic nematode community of moose across a wide latitudinal range. Overall, the population-specific impact of migration on parasitism across the distribution range and variation in sympatry with other ruminants suggest local characteristics affect host-parasite relationships.

## Highlights

• Characterized the nematode community of moose across 11° of latitude by DNA metabarcoding.
• Detected 21 nematode taxa including 7 species of livestock parasites in wild moose.
• Host-population specific effects of migration on nematode richness and prevalence.
• Nematode richness was higher in winter than in summer.
• Local characteristics were more important than latitudinal gradient in explaining nematode alpha diversity.

## Graphical abstract

**Figure:**
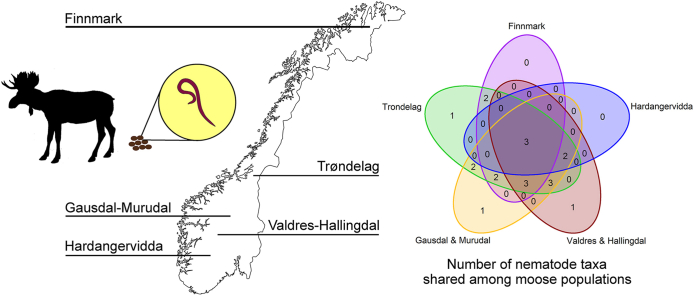

## 1 Introduction

Parasitic nematodes are ubiquitous throughout nature and can have significant negative effects on their host (Hoberg et al., 2001). Indeed, some nematode species can cause reduced body condition and fecundity or increased mortality (Gulland, 1992; Gunn and Irvine, 2003), but many species may actually be benign (Leung and Poulin., 2008). Nematode communities vary geographically with variation in infestation among both individuals and populations (Wells et al., 2015; Albery et al., 2022), and alpha diversity often follows a general latitudinal species gradient with more species found near the equator (Poulin, 2014; Preisser, 2019). Baseline knowledge of parasite biodiversity is one essential component for anticipating and detecting altered patterns of geographic distribution of parasitic infections and diseases (Kutz et al., 2004). While the nematode fauna is well described in most livestock species, comparatively less is known in wild ruminants, particularly in northern latitudes (Kutz et al., 2004; Domke et al., 2013; Rose et al., 2014). Studies from wildlife are often based on a limited sample size or are derived from a single population (Davidson et al., 2014; Filip-Hutsch et al., 2021). This partly reflects methodological limitations with time consuming egg and larvae-counts often relying on fresh fecal samples which are more difficult to collect from wildlife than domestic animals. With the advent of molecular-based assay methods, there are novel opportunities to screen large numbers of samples from multiple populations with standardized techniques (Beaumelle et al., 2021; Davey et al., 2023). It should also provide higher taxonomic resolution than traditional egg flotation methods (Davey et al., 2023), although issues regarding infection intensity estimates are likely to remain (Eysker and Ploeger, 2000; Davidson et al., 2015).

Patterns of infection across wildlife hosts are affected by both exposure processes and host suitability. Animals become infected with environmentally transmitted nematodes through passive ingestion of larva and eggs when feeding or when larva burrow through the skin (Anderson, 2000). Therefore, individual foraging and habitat use patterns determine exposure risk and are an important factor affecting infection patterns (Ezenwa, 2004; Ezenwa et al., 2016). For example, preferentially spending time in areas rich in resources that attract many individuals may increase the risk of infection to a wider range of nematode species through host aggregation (Wright and Gompper, 2005; Utaaker et al., 2023). However, even within species not all hosts are equally susceptible. Juveniles of long lived animals lack acquired immunity while males can experience testosterone suppressed immune function, often resulting in these two demographics being most prone to infection (Zuk, 2009; Body et al., 2011). Further, due to temperature and moisture tolerance of nematode eggs and larva in the environment, their survival and growth can vary among different habitats and across seasons (Stien et al., 2002; Albery et al., 2018; Peacock et al., 2022), generating geographic and temporal variation in infection rates.

Migration involving broad seasonal movements between disparate ranges at a landscape level and its relationship with parasite infection has gained much interest over the past decade (Altizer et al., 2011; Binning et al., 2022). Seasonal movement away from wintering grounds contaminated with large quantities of parasites such as ticks, warble flies, or protists allow individuals to temporarily escape infection pressure (Folstad et al., 1991; Mysterud et al., 2016; Slowinski et al., 2018). However, there have been contradictory findings of migratory animals harboring higher parasite richness or having higher prevalence of specific parasite species than non-migratory animals, possibly due to higher encounter rates or increased susceptibility to infection (Figuerola and Green, 2000; Koprivnikar and Leung, 2015; Teitelbaum et al., 2018). Notably, most studies investigating migration behavior and nematode infection have been conducted in fish and birds (Balling and Pfeiffer, 1997; Koprivnikar and Leung, 2015) while few have looked at similar patterns in wild mammals (Teitelbaum et al., 2018; Vestbo et al., 2019), thereby limiting our understanding of the effect of migratory behavior on parasitism.

**Fig. 1:**
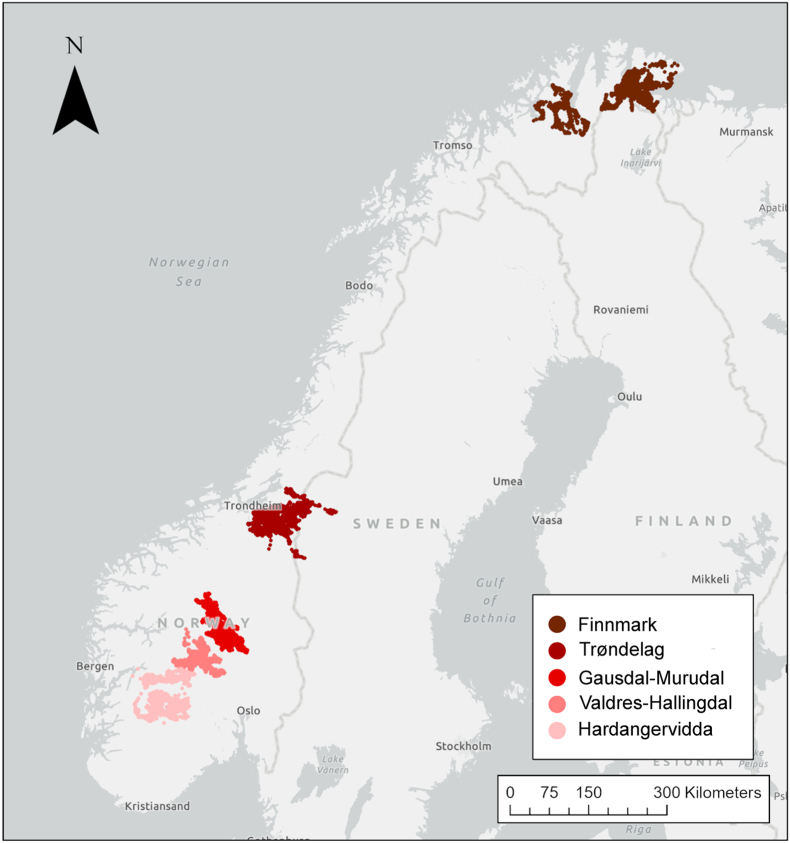
A map showing the distribution of the five study areas across Norway ranging from 59.6°N to 70.5°N.

Moose (*Alces alces*) are widely distributed throughout the northern coniferous forests and represent an ideal system for studying how parasite diversity and prevalence is structured through the interplay of individual and population level characteristics across wide latitudinal gradients that encompass diverse habitats and environments. In this study, we utilized DNA metabarcoding of fecal samples (n = 264) to characterize the nematode community of GPS marked moose (n = 235) from five populations distributed across a large geographic area in Norway ranging from the south to far north of the arctic circle (Fig. 1, Table 1). We first determined how the nematode community varied geographically throughout Norway with a particular focus on spillover of parasites from domestic ungulates (i.e. sheep, goats, and cattle), and tested if there was a predictable latitudinal gradient in alpha diversity. Demographically we expected a higher nematode richness in males compared to females, and in young compared to adult moose. We subsequently tested if moose harbored higher nematode richness in summer as compared to winter. Lastly, we investigated if higher proportional use of specific habitat types (e.g. deciduous forest or open areas) and seasonal migration were associated with higher nematode richness and prevalence, and whether any observed trends were consistent across all populations.

**Table 1:** Characteristics of moose home ranges at each study area where migration distance, elevation change, and the densities of both wild and domestic ruminants are averaged across all individuals in each study area.

Region	Finnmark	Trøndelag	Gausdal-Murudal	Valdres-Hallingdal	Hardangervidda
Sample years	2016–2018	2017–2022	2020–2021	2014–2015	2021
Mean Latitude	70.2	63.2	61.3	60.7	60.1
Elevation range (m)	1–375	136–821	465–1168	426–1072	891–1278
Avg. winter range elevation (m)	87	422	868	629	1110
Avg. summer range elevation (m)	169	512	813	854	1113
Avg. migration distance (km) (range)	18.88 (1.37–59.64)	9.26 (0.22–58.78)	48.64 (1.15–119.37)	13.22 (0.18–52.16)	11.74 (1.26–49.69)
Avg. elevation change (m) (range)	81.94 (−134.27 - 27.41)	90.17 (−230.8 - 372.02)	−54.7 (−411.11 - 321.51)	225.01 (−197.77 - 592.28)	3.17 (−182.31 - 178.62)
Harvest density of moose/km2	0.106	0.353	0.244	0.248	0.176
Harvest density of red deer/km2	0.000	0.058	0.124	0.056	0.185
Harvest density of roe deer/km2	0.000	0.158	0.092	0.110	na
Density of reindeer/km2	2.581	1.662	1.725	0.848	0.835
Density of dom. Sheep/km2	11.05	4.6	13.95	87.23	2.49
Density of cattle/km2	0.00	0.09	0.36	<0.01	<0.01

## 2 Methods

### 2.1 Study areas

Data were collected from five different study areas distributed along a south-north gradient from 59.6°N to 70.5°N (decimal degrees) in Norway (Fig. 1, Table 1). All areas are found within the boreal and alpine vegetation zones (Moen, 1999), but span a large variety of habitats, altitudes, and climates. Forests throughout the latitudinal gradient are dominated by Norway spruce (*Picea abies*), Scots pine (*Pinus sylvestris*) and downy birch (*Betula* spp.), except for the most northern population in Finnmark where spruce is absent and pine is scarce. In addition, grey alder (*Alnus incana*), rowan (*Sorbus aucuparia*), aspen (*Populus tremula*), sallow (*Salix caprea*), other willows (*Salix* sp.) and juniper (*Juniperus communis*) are present at lower densities in all areas. The altitude woodland limit (i.e., highest group of trees) ranges from about 1000 to 1100 m.a.s.l. in the south (Gausdal-Murudal, Valdres-Hallingdal, Hardangervidda) to less than 100 m.a.s.l. in northern parts of Finnmark (Moen, 1999).

In all study areas winters are long and moderately cold, with snow covering the whole or significant parts of the land for approximately 6–9 months of the year (Fig. S1), but due to local conditions, elevations, and annual variations certain sub-areas may be covered in snow for significantly shorter time periods. Particularly long winters are found in Finnmark and Hardangervidda, as well as at higher altitudes in all study areas. Summers are rather cool in all of Norway, particularly at higher altitudes (Fig. S1). Farming is conducted within the lowland perimeters of all study areas, but to a lesser extent in Finnmark and Hardangervidda. Farmlands are mainly used for grazing sheep and cattle (dairy), as well as for production of livestock winter fodder (grass). In addition, free ranging sheep (and to a lesser extent cattle) at varying densities are grazing in forests and alpine pastures during the summer season in all study areas (Table 1).

### 2.2 Fecal sampling

Fecal samples were collected from the rectum of 235 moose that were captured and GPS-collared during February and March 2014–2021 (Table 1). Moose were first located from a helicopter and subsequently immobilized using etorphine injected by dart gun. We equipped each moose with a GPS-collar with a VHF–beacon (Vectronic Aerospace GmbH, Berlin, Germany) as well as a numbered plastic tag in each ear. All procedures were approved by the Norwegian Animal Research Authorities (reference numbers: 2015/225449, 18/22533, 20/227896, 22/254339, 2013/216214, & 19/254723). For 29 females in Trøndelag and Gausdal-Murudal, an additional fecal sample was collected in June–August when checking the status of their calves. We used the VHF-beacon to approach and observe the number of calves following the collared mother, and when possible, collected fecal samples from the ground where the moose was observed. The samples were stored in ethanol or silica in the field, and when returning from fieldwork they were stored at −20 °C for a few days followed by −80 °C at the Norwegian Institute for Nature Research (NINA) in Trondheim Norway until DNA extraction occurred.

### 2.3 Extraction, PCR, and metabarcoding

DNA extractions and library construction were carried out at the Center for Biodiversity Genetics (NINAGEN) at NINA. Fecal samples were placed in 2 mL homogenization tubes containing lysing matrix E (blend of 1.4 mm ceramic spheres, 0.1 mm silica spheres, and one 4 mm glass bead; MP biomedicals), 980 μL phosphate buffer and 122 μL MT lysis buffer (MP Biomedicals). Samples were homogenized for 1 min at 1600 rpm using a FastPrep-96™ instrument (MP Biomedicals), then centrifuged for 10 min at 11,500 rpm. A 400 μL subsample of the supernatant was then transferred to the sample plate of a KingFisher MagMAX Microbiome Ultra Nucleic Acid Isolation Kit (ThermoFisher Scientific). DNA was then isolated using a KingFisher Flex automated extraction instrument (Thermo Fisher Scientific Inc.) following the manufacturer's standard protocol. PCR amplification of the ITS2 region of rDNA was carried out following Davey et al. (2023) using primers NC1 (forward) and NC2 (reverse) designed specifically for clade V parasitic nematodes (Gasser et al., 1993). The PCR products were visualized and quantified using Nanodrop (ThermoFisher Scientific) and TapeStation 4200 systems (Agilent) before being cleaned using magnetic beads (Mag-Bind RxnPure Plus) and thereafter conducting a second indexing PCR using IDT for Illumina UD index kits (Illumina, USA) following the manufacturer's instructions. After a 2nd cleaning of the indexed samples using magnetic beads, amplicons were pooled in equimolar amounts and sequenced in a single paired-end 250 bp run (NovaSeq SP full flow cell) on an Illumina NovaSeq 6000 instrument at the Norwegian Sequencing Center (NSC), University of Oslo, Norway. Sequences are deposited in the Sequence Read Archive (SRA; accession number PRJNA1129464).

### 2.4 Bioinformatics

Primer sequences were removed from both the forward and reverse reads using cutadapt v.3.5, with an allowed 15% mismatch (Martin, 2011). Quality filtering, error correction, and chimera detection and removal were further conducted in R using DADA2 v26.0 (Callahan et al., 2016; Team, 2022). Sequences with ambiguous bases, >2 expected errors in either the forward or reverse reads, and <50bp overall length were removed. Because Illumina bins error rates in order to more efficiently handle the large quantity of data produced by NovaSeq, we estimated error rates by enforcing monotonicity for both the forward and reverse reads. Sequence variants were inferred for each sample and the forward and reverse reads were merged with a minimum overlap of 30 bp. Finally, chimeric sequences were assessed on a per-sample basis, as chimeras are formed at the individual PCR-level. Any sequence variants flagged as chimeric in more than 90% of the samples they occurred in were removed from the dataset.

Taxonomy was assigned to all amplicon sequence variants (ASVs) using the IDTaxa function in the DECIPHER package v. 2.26.0 (Wright, 2016) with a customized version of the ITS2 Nemabiome database (http://www.nemabiome.ca) including additional strongylid reference sequences as described in Davey et al. (2023). We also conducted a megablast search (Morgulis et al., 2008) against the NCBI nucleotide non-redundant database (Sayers et al., 2022). ASVs were considered as target nematode taxa when identified with greater than 60% confidence using IDTaxa and a best BLAST match of more than 90% identity and 80% coverage to a nematode reference sequence. All non-target sequences were removed, and final taxonomic assignments were made based on the lowest taxonomic level receiving a >60% confidence score in the IDTaxa analysis. Any ASVs with <10 sequences in the dataset were excluded from further analyses. The PCR no-template negative control contained just 88 sequence reads after quality filtering. Because such minimal contamination was assumed to be negligible across the dataset with little effect on downstream analyses, no measures were taken. ASVs were clustered into operational taxonomic units (OTUs) with a 97% sequence similarity threshold in the IdCluster algorithm from the DECIPHER package with each OTU receiving the taxonomic assignment of its most abundant ASV (Wright, 2016). Finally, because our main interest is nematode species and community level trends rather than genetic diversity, the OTUs were collapsed to the lowest level taxonomy assigned (e.g. species or genus). If a resulting genus contained more than one OTU it is listed as “spp.” (Table S1).

### 2.5 Habitat-use and migration data

To determine if the use of different habitat types affects the nematode community composition in moose, we calculated the proportional use of each type of land cover from the AR5 map of Norway (Ahlstrøm et al., 2019) for each individual based on the proportional number of GPS reads across an entire year starting in April and ending in March the following year. The AR5 distinguishes cultivated land, pastures, open land, marsh land, fresh water, sea, glacier, built-up land, and forests. Forests are distinguished based on the dominant tree species (coniferous, broad-leaf, or mixed forest) and their productivity (very low, low, middle, high, and very high). For 130 individuals with multiple years of GPS data, we calculated the among year variance in proportional habitat-use variables to account for the GPS data being collected after fecal sampling in order to remove bias. The variance for all variables for all individuals was <0.001 indicating similar among year habitat use patterns. Therefore, the first full year of GPS data following sampling was used for our models.

The net squared displacement approach was used to calculate all variables related to migration such as migration status (i.e. resident or migrant) and migration distance among others (Bunnefeld et al., 2011). Because migratory status could not be reliably determined for all individuals, we instead used distance between center point of summer and winter home range (hereafter termed migration distance).

To determine if GPS data being collected after initial sampling is an adequate description of space use, we tested the among year repeatability of mean summer range elevation, mean winter range elevation, and log transformed migration distance (for normalization) of the 127 individuals with multiple full years of migration data. This was conducted using intraclass correlation coefficient (ICC) by LMM method in the “rptR” package with 1000 boot straps where individual ID was the random effect (Nakagawa and Schielzeth, 2010). We found that both mean winter (r = 0.95, SE = 0.009, *p* < 0.001) and summer (r = 0.98, SE = 0.003, *p* < 0.001) elevations were highly repeatable and migration distance was reliably repeatable (r = 0.67, SE = 0.041, *p* < 0.001). We then calculated the change in elevation by subtracting mean winter elevation from mean summer elevation.

### 2.6 Statistical analysis

#### 2.6.1 Geographic model and seasonality

To test for variation in nematode taxa alpha diversity among study areas we utilized a linear mixed effects model (LMM) from the “lme4” package combined with “lmerTest” where nematode taxonomic richness was the response variable (Bates et al., 2015; Kuznetsova et al., 2017). We included collection year as a random effect with sex, age class (i.e. calf, yearling, adult, unknown), and study area as fixed effects. The model was checked for goodness of fit by plotting the residuals on a quantile-quantile plot and the variance inflation factor (VIF) was used to determine that there was no multicollinearity among the explanatory variables. This led to the exclusion of ungulate densities from the final model. Using the “multcomp” package we then conducted Tukey's post-hoc analyses on both age class and study area to gain further insights into how nematode taxonomic richness varies amongst them (Hothorn et al., 2008). To test for the marginal effect of sex, age class, collection year, and study area on betadiversity, we used the “adonis2” function from the R package “vegan” to run a PERMANOVA with the function margin = “by” (Oksanen et al., 2022). Three individuals with no nematodes detected were excluded from the analysis because dissimilarity values could not be calculated for them.

To explore variation in nematode prevalence across Norway, we used a generalized linear model (GLM) with binomial distribution (package “lme4”; Bates et al., 2015) run separately for five of the six most prevalent nematodes identified (i.e. *Nematodirella* spp., *Elaphostrongylus alces*, *Trichostrongylus axei*, *Trichostrongylus* spp. and the unidentified Strongylida). *Ostertagia* spp. was excluded because most animals were infected with this nematode (97.9% prevalence), although it may comprise several species known to parasitize moose (Davidson et al., 2015). We included the same variables as the richness model but excluded year due to the model not converging if included as a random effect and moderate multicollinearity between year and study area if included as a fixed effect. We then utilized Tukey's post-hoc analyses for pairwise comparisons of nematode prevalence among the five study areas as well as among host age class.

Using a zero-inflated Gaussian mixed model with the R package NBZIMM, we tested for differences in nematode richness between summer and winter (Zhang et al., 2018). We only included 29 individuals with samples collected in both seasons. The random effect was individual ID and fixed effects were study area and season.

#### 2.6.2 Habitat use

We used a separate LMM to test the effect of proportional use of different habitat types on nematode taxa richness because not all individuals had adequate GPS data for estimating habitat use patterns. Only those individuals with at least one full year of GPS data were included in our models (n = 194 individuals). To avoid multicollinearity, we excluded variables with a high degree of correlation (−0.7 ≥ r ≥ 0.7). Variables were excluded in a stepwise fashion where those with the highest number of correlations were removed followed by the second and so on until all r values were between −0.7 and 0.7. In addition, we used VIF on our models to confirm there were no issue of multicollinearity. The global model presented here includes host sex, age class, and the proportional use of deciduous forest, mixed forest, agricultural areas, grazing pastures, marsh land, and open land with year and study area as random effects. A PERMANOVA with jaccard dissimilarity matrix was used to test the marginal effects of the same variables on beta-diversity with year and study area also included. We could not directly compare the proportional use of different habitat types in relation to nematode taxa richness through interaction terms in the global model due to the high among study area variability in habitat structure and use patterns causing a large increase in model complexity (based on AIC). Therefore, we analyzed the effect of proportional habitat use on both alpha and beta nematode diversity at each study area separately using a linear model (LM) or PERMANOVA with Jaccard dissimilarity respectively. Year was excluded from the models due to singularity issues, but individual age class and sex were included. Furthermore, because each study area varies in habitat structure, we excluded proportional use variables which accounted for <3% of habitat use on average among individuals at each location.

To test the effect of habitat use patterns on prevalence of five out of the six most common nematode taxa, excluding *Ostertagia* spp. due to near 100% prevalence, we ran Firth's bias-reduced logistic regression with presence/absence as a binomial response variable for each nematode separately across all study areas (i.e. global model). Age class, study area, and the proportional use of each habitat type were included as fixed effects. We then ran models for each study area separately with age class, sex, and proportional use of each habitat variable as fixed effects. Similar to our study area specific richness models, we excluded proportional habitat use variables that account for on average < 3% at each site. Despite the simplified model, it often failed to converge when nematode prevalence was low (about 10% or lower) at a specific study area.

#### 2.6.3 Migration

Similar to the habitat use model, we ran a separate model analyzing the effect of migration behavior on nematode taxa richness including only those individuals for which a full year of GPS data had been collected in order to allow accurate determination of winter and summer home ranges (n = 191 individuals). We utilized a LMM with sampling year as a random variable to test the effect of host sex, age class, study area, elevation change, and log-transformed migration distance on nematode taxa richness. We also included interaction terms for study area with log-transformed migration distance and elevation change. Finnmark was chosen as the reference value for study area due to both migration distance and elevation change having minimal effect on nematode taxa richness in this study area, thereby serving as a base comparison for all other areas. The effect of the same variables on beta diversity with year also included was tested for using PERMANOVA with jaccard dissimilarity matrix. We tested for their conditional effects rather than marginal to allow for both the main effect and interaction terms to be included. Using Firth's bias-reduced logistical regression we tested the effect of host sex, age class, study area, log-transformed migration distance, elevation change, and two separate interaction terms for study area with log-transformed migration distance and elevation change on prevalence of five of the six most common nematodes (*Ostertagia* spp. was excluded). Year was excluded from the models due to lack of convergence. Furthermore, the interaction of study area and elevation change was excluded from the model for *E. alces* due to lack of convergence and the interaction of study area with log-transformed migration distance was excluded from the model for the unclassified Strongylida due to separation of data.

## 3 Results

### 3.1 Parasite taxa and metabarcoding

A total of 146 OTUs consisting of 2996 ASVs containing 69,319,984 sequence reads were recovered post-quality filtering and combined into 21 nematode taxa (Table 2, Table 3). The six most common nematode taxa found in winter among all individuals were *Ostertagia* spp. (97.9% prevalence), unclassified Strongylida (60.4%), *Nematodirella* spp. (59.6%), *Trichostongylus* spp. (17.4%), *Trichostrongylus axei* (11.5%), and *Elaphostrongylus alces* (11.5%). All remaining taxa exhibited less than 10% prevalence although it varied among study areas (Table 2).

**Table 2:** Prevalence of each nematode taxa in each study area at time of marking (winter). Species names in bold are also found in domestic sheep and goats in Norway (Domke et al., 2013). Other taxa such as *Bunostomum* and *Cooperia* are also found in sheep though the species could not be determined.

	Finnmark	Trøndelag	Gausdal-Murudal	Valdres-Hallingdal	Hardangervidda	Total
Nematode taxa	n = 52	n = 81	n = 44	n = 38	n = 20	n = 235
*Bunostomum* sp.	9.6	3.7	0.0	0.0	0.0	3.4
***Chabertia ovina***	0.0	2.5	4.5	2.6	0.0	2.1
*Chabertia* sp.	0.0	0.0	2.3	0.0	0.0	0.4
*Cooperia* spp.	0.0	6.2	2.3	0.0	0.0	2.6
*Elaphostrongylus alces*	0.0	18.5	13.6	10.5	10.0	11.5
*Elaphostrongylus* spp.	1.9	4.9	0.0	0.0	0.0	2.1
***Haemonchus contortus***	0.0	2.5	20.5	5.3	0.0	5.5
*Nematodirella* spp.	53.8	56.8	63.6	55.3	85.0	59.6
*Ostertagia gruehneri*	0.0	1.2	0.0	0.0	0.0	0.4
*Ostertagia ostertagi*	0.0	8.6	6.8	0.0	0.0	4.3
*Ostertagia* spp.	100.0	97.5	97.7	94.7	100.0	97.9
*Spiculopteragia boehmi*	7.7	7.4	2.3	0.0	0.0	4.7
*Spiculopteragia* spp.	19.2	7.4	2.3	0.0	0.0	7.2
***Teladorsagia circumcincta***	0.0	6.2	6.8	2.6	0.0	3.8
***Trichostrongylus axei***	5.8	12.3	27.3	5.3	0.0	11.5
***Trichostrongylus colubriformis***	0.0	7.4	27.3	5.3	5.0	8.9
*Trichostrongylus* spp	5.8	32.1	18.2	10.5	0.0	17.4
***Trichuris ovis***	0.0	0.0	0.0	2.6	0.0	0.4
Unclassified 1 (Haemonchidae)	11.5	1.2	2.3	2.6	0.0	3.8
Unclassified 2 (Strongylida)	84.6	64.2	68.2	36.8	10.0	60.4

**Table 3:** Prevalence of nematode taxa detected in winter and summer samples collected from 29 female moose from two study areas. Species names in bold are also found in domestic sheep and Goats in Norway (Domke et al., 2013). Other taxa such as *Cooperia* spp. and *Trichostrongylus* spp. are also found in sheep and goats though the species could not be determined.

	Trøndelag		Gausdal-Murudal		Total	
Nematode	Winter (N = 19)	Summer (N = 19)	Winter (N = 10)	Summer (N = 10)	Winter (N = 29)	Summer (N = 29)
*Cooperia* spp.	5.3	0.0	0.0	0.0	3.4	0.0
*Elaphostrongylus alces*	5.3	0.0	0.0	0.0	3.4	0.0
*Elaphostrongylus* spp.	5.3	0.0	0.0	0.0	3.4	0.0
***Haemonchus contortus***	5.3	10.5	0.0	10.0	3.4	10.3
*Nematodirella* spp.	52.6	15.8	50.0	0.0	51.7	10.3
***Oesophagostomum venulosum***	0.0	5.3	0.0	0.0	0.0	3.4
*Ostertagia gruehneri*	0.0	5.3	0.0	0.0	0.0	3.4
*Ostertagia ostertagi*	5.3	0.0	0.0	0.0	3.4	0.0
*Ostertagia* spp.	94.7	31.6	90.0	10.0	93.1	24.1
*Spiculopteragia boehmi*	0.0	5.3	0.0	0.0	0.0	3.4
*Spiculopteragia* spp.	5.3	5.3	0.0	0.0	3.4	3.4
***Trichostrongylus axei***	5.3	5.3	30.0	0.0	13.8	3.4
***Trichostrongylus colubriformis***	15.8	0.0	20.0	10.0	17.2	3.4
*Trichostrongylus* spp	42.1	5.3	20.0	0.0	34.5	3.4
Unclassified 2 (Strongylida)	47.4	26.3	60.0	10.0	51.7	20.7

### 3.2 Individual and population level characteristics

**Fig. 2:**
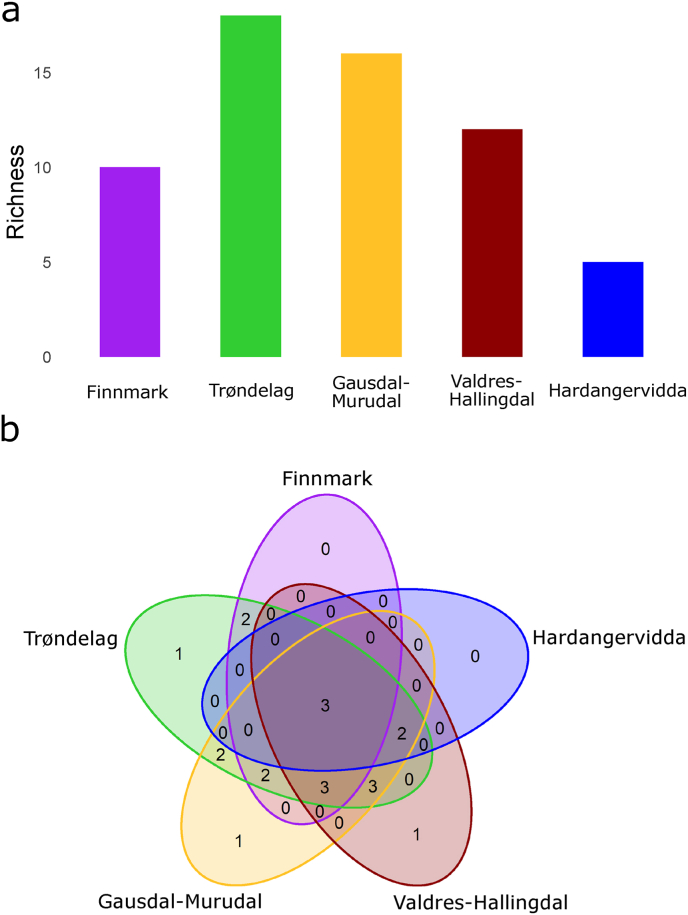
Nematode component community in winter with a) the number of nematode taxa detected at each study area and b) the number of nematode taxa shared among study areas.

The highest number of nematode taxa were found in Trøndelag and the lowest in Hardangervidda with 18 and five taxa respectively (Fig. 2a). Only three nematode taxa (*Ostertagia* spp., *Nematodirella* spp., and the unclassified Strongylida) were detected across all study areas (Fig. 2b). *Chabertia* sp., *Oesophagostomum venulosum*, *Ostertagia gruehneri*, and *Trichuris ovis* were each found in only one study area (Trøndelag, Gausdal-Murudal, and Valdres-Hallingdal respectively) with very low prevalence (Fig. 2b, Table 2, Table 3). The northernmost study area (i.e. Finnmark) and the southernmost (i.e. Hardangervidda) shared the least number of nematode taxa (three in total) while Trøndelag and Gausdal-Murudal shared the highest with 15 (Fig. 2b).

We found that individuals harbored significantly lower nematode richness in Hardangervidda (LMM: b = −1.037, SE = 0.506, *p* = 0.045) as compared to Finnmark (Table S2) and Gausdal-Murudal (Tukey's post-hoc: b = −1.041, SE = 0.350, *p* = 0.021; Table S3). There was no significant difference between the other study areas (Tables S2 and S3). We further found that calves harbored higher nematode richness as compared to adults although it was marginally non-significant (LMM: b = 0.732, SE = 0.373, *p* = 0.052; Table S2). No difference was found between yearlings and adults (LMM: b = 0.384, SE = 0.337, *p* = 0.255) or yearlings and calves (Tukey's post-hoc: b = −0.348, SE = 0.478, *p* = 0.879; Fig. S2, Tables S2 and S3). There was a significant effect of study area on beta-diversity (PERMANOVA: SS = 2.507, R^2^ = 0.093, *p* = 0.001) in addition to host age class (PERMANVOA: SS = 0.843, R^2^ = 0.031, *p* = 0.001), but not collection year (PERMANOVA: SS = 0.164, R^2^ = 0.006, *p* = 0.160; Table S4). No effect of sex was found on either nematode taxa richness (LMM: b = 0.132, SE = 0.178, *p* = 0.462) or beta diversity (PERMANOVA: SS = 0.120, R^2^ = 0.004, *p* = 0.323; Tables S2 and S4).

**Fig. 3:**
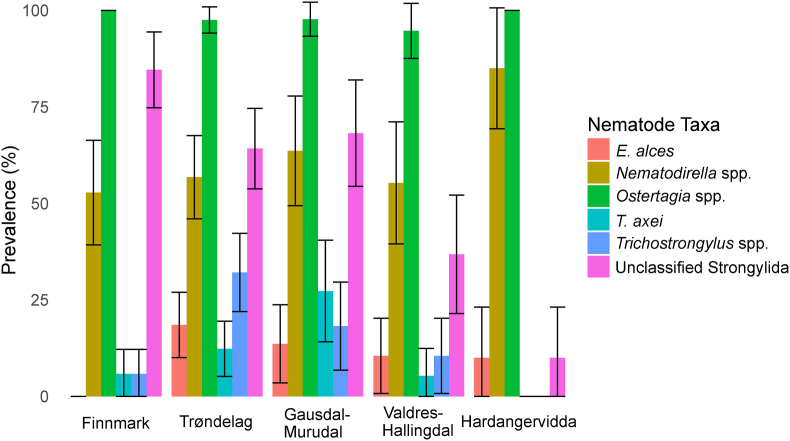
Prevalence in each study area of the six most common nematodes detected. Whiskers indicate 95% confidence intervals.

For the five nematodes we tested for differences in prevalence among study areas, we found significantly higher prevalence of *Nematodirella* spp. in Hardangervidda as compared to Finnmark (GLM: b = 1.599, SE = 0.698, *p* = 0.022), *T. axei* in Gausdal-Murudal than in Finnmark (GLM: b = 1.768, SE = 0.715, *p* = 0.013), and *Trichostrongylus* spp. in Trøndelag than in Finnmark (GLM: b = 2.29, SE = 0.663, *p* < 0.001; Fig. 3, Table 2 and S5). There was also a significantly lower prevalence of *Trichostrongylus* spp. in Valdres-Hallingdal than in Trøndelag (Tukey's post-hoc: b = −1.6, SE = 0.601, *p* = 0.045; Fig. 3, Table S6). Prevalence of the unclassified Strongylida was significantly lower in Hardangervidda (GLM: b = −3.859, SE = 0.847, *p* < 0.001) and Valdres-Hallingdal (GLM: b = −2.133, SE = 0.517, *p* < 0.001) as compared to Finnmark (Fig. 3, Table 2 and S5), Valdres-Hallingdal than in Trøndelag (Tukey's post-hoc: b = −1.273, SE = 0.446, *p* = 0.032) and Gausdal-Murudal (Tukey's post-hoc: b = −1.346, SE = 0.485, *p* = 0.041), and Hardangervidda than in Gausdal-Murudal (Tukey's post-hoc: −3.073, SE = 0.82, *p* = 0.002; Fig. 3, Table 2, S5, and S6). There was significantly higher prevalence of *Nematodirella* spp. (GLM: b = 1.895, SE = 0.698, *p* = 0.007) in calves as compared to adults, but no effect of age category was found for any other taxa (Tables S5 and S7).

Nematode taxa richness was significantly higher in winter than in summer (ZIGMM: b = 1.117, SE = 0.417, *p* = 0.012) with no nematode DNA found in 19 of 29 summer samples. Richness was similar between study areas (ZIGMM: b = 0.623, SE = 0.416, *p* = 0.145). The nematodes *Ostertagia* spp., *Nematodirella* spp., and *Trichostrongylus* spp. were found to have higher prevalence in winter (93.1%, 51.7%, and 34.5%, respectively) as compared to summer (24.1%, 10.3%, 3.4% respectively; Table 3). Most other nematode taxa showed a similar trend but to a lesser extent (Table 3). The two exceptions were the sheep parasites *Oesophagostomum venulosum* that was only found in one individual in summer and *Haemonchus controtus* with prevalence three times higher in summer (10.3%) than in winter (3.4%; Table 3).

### 3.3 Habitat use

In the global habitat use model there was a significant positive effect of the proportional time spent in grazing pastures on nematode richness (LMM: b = 14.404, SE = 7.188, *p* = 0.047), but not beta-diversity (PERMANOVA: SS = 0.175, R^2^ = 0.005, *p* = 0.346; Tables S8 and S9). There was no significant effect of time spent in any other habitat type on nematode richness or community composition in the global model nor in the study area specific models (Tables S8–S11). Calves had significantly higher nematode richness in both the global (LMM: b = 1.047, SE = 0.38, *p* = 0.007) and in the Trøndelag specific (LM: b = 1.272, SE = 0.413, *p* = 0.003) models, but no effect in any other study area (Tables S8 and S10). Age class similarly had an effect on beta-diversity in the global model (PERMANOVA: SS = 0.979, R^2^ = 0.029, *p* = 0.003) and in the Trøndelag specific model (PERMANOVA: SS = 1.243, R^2^ = 0.102, *p* = 0.002; Tables S9 and S11). There was no effect of sex on nematode richness in either the global model (LMM: b = 0.259, SE = 0.19, *p* = 0.176) or study area specific models (all p > 0.05; Tables S8 and S10). Furthermore, only in the Trøndelag model was a significant effect of sex found on beta-diversity (PERMANOVA: SS = 0.518, R^2^ = 0.043, *p* = 0.009; Table S11).

There was no effect of proportional use of any habitat type on prevalence of the five nematode taxa tested in either the global or study area specific models (Tables S12 and S13). Calves had a significantly higher prevalence of *Nematodirella* spp. in both the global (Firth's: b = 1.335, SE = 0.685, *p* = 0.041) and Trøndelag specific (Firth's: b = 1.883, SE = 0.803, *p* = 0.011) models (Tables S12 and S13). We also found significantly higher prevalence of *E. alces* in calves (Firth's: b = 2.337, SE = 0.709, *p* = 0.001) and yearlings (Firth's: b = 2.527, SE = 0.686, *p* = 0.001) in the global model as well as the Trøndelag specific model (Firth's: calves: b = 3.445, SE = 0.985, *p* < 0.001; yearlings: b = 4.101, SE = 1.344, *p* = 0.002; Tables S12 and S13). Males were found to harbor significantly higher prevalence of *E. alces* in the global model (Firth's: b = 1.047, SE = 0.49, *p* = 0.043) but significantly lower prevalence of *Trichostrongylus* spp. (Firth's: b = −1.598, SE = 0.714, *p* = 0.017) in the Trøndelag specific model (Tables S12 and 13).

### 3.4 Migration

There was a significant positive effect of log-transformed migration distance on nematode taxa richness at both Gausdal-Murudal (LMM: b = 0.848, SE = 0.205, *p* < 0.001) and Trøndelag (LMM: b = 0.389, SE = 0.169, *p* = 0.023; Table S14). There was also a significant positive effect of elevation change on nematode taxa richness at Gausdal-Murudal (LMM: b = 0.006, 0.002, *p* = 0.013), although the effect size is notably small (Table S14). The main effect of migration distance significantly effected beta-diversity (PERMANOVA: SS = 0.612, R^2^ = 0.018 *p* = 0.003) but not its interaction with study area (PERMANOVA: SS = 0.721, R^2^ = 0.022, *p* = 0.274; Table S15).

When exploring the effect of migration behavior on the prevalence of individual nematode taxa, there was a significant positive effect of log-transformed migration distance on infection with *Nematodirella* spp. (Firth's: b = 1.055, SE = 0.438, *p* = 0.01) and *T. axei* (Firth's: b = 2.563, SE = 0.921, *p* = 0.008) at Gausdal-Murudal as well as *E. alces* at Valdres-Hallingdal (Firth's: b = 1.443, SE = 0.731, *p* = 0.037; Table S16). The only effect of elevation change was a significantly positive effect on infection with *Trichostrongylus* spp. (Firth's: b = 0.015, SE = 0.007, *p* = 0.042) at Gausdal-Murudal (Table S16).

## 4 Discussion

Wildlife in northern ecosystems are regarded as particularly sensitive to parasitic infections under global climate change (Kutz et al., 2013), yet we lack an in-depth baseline of nematode diversity and infection prevalence as well as their drivers for many species (Rose et al., 2014). DNA metabarcoding now provides the opportunity to survey the parasitic nematode community across sufficiently large sample sizes to allow inferences about both individual and population level variation across wide latitudinal gradients and environmental conditions. Our study is the first broad scale description of nematode diversity and prevalence in moose, the most economically important game species in Norway, Sweden, and Finland (Lavsund et al., 2003). Using DNA metabarcoding, we detected 21 different nematode taxa including several that more typically parasitize sheep (e.g. *Haemonchus contortus* and *Trichuris ovis*). There was no clear latitudinal gradient in the nematode community, and the effect of migration on parasitism was not consistent across populations. The use of DNA metabarcoding on our large sample size derived from individually GPS-marked moose shows how local factors affect geographic patterns of nematode diversity.

### 4.1 Geographic variation in the nematode and host communities

Wildlife in northern ecosystems may have a sparser parasite community than those inhabiting tropical or temperate ecosystems following a classic latitudinal diversity gradient. However, unequal sampling among different global regions complicates determining such patterns (Hoberg et al., 2012; Dallas et al., 2018). The lack of an alpha diversity gradient across 10 degrees of latitude in our study suggest that local factors such as climate, host behavior, density, and overlap with both wild and domestic ungulates are more important than latitude in explaining the among population differences in the nematode communities and the taxa detected (Fig. 2, Table 2, Table 3). How this compares to the most southern moose populations in Europe is uncertain as few studies on the individual host level parasite communities have been conducted (Świslocka et al., 2020).

Red deer, roe deer, and (wild and semi-domestic) reindeer occupy some of the same habitat as moose in Norway (Table 1), and the level of habitat sharing may determine exposure risk of moose to novel parasitic nematode species (Dougherty et al., 2018; Świsłocka et al., 2021). We detected multiple species of nematodes with low prevalence for which moose are likely non-ideal hosts such as the parasite *Ostertagia gruehneri* or *Spiculopteragia boehmi* that are more typically found in reindeer, red deer, and roe deer (Manninen et al., 2014; Demiaszkiewicz et al., 2017; Pyziel-Serafin et al., 2023). The presence of other ungulate species likely increases exposure of moose to nematodes for which they cannot maintain within their own population. Indeed, the highest number of nematode taxa detected were in the three study areas (i.e. Trøndelag, Gausdal-Murudal, and Valdres-Hallingdal) where all four deer species are present (Fig. 2). A similar finding was reported in Poland where the nematode *Elaphostrongylus cervi* was found in moose after the establishment of red deer, the main host, in the Biebrza valley (Świslocka et al., 2020; Świsłocka et al., 2021). As climate change is expected to facilitate a north and eastward expansion of red deer distribution in Norway (Rivrud et al., 2019), moose in these areas may be at increased risk of exposure to novel parasites.

In addition to nematode sharing among wild ungulates, spillover from domestic ruminants such as sheep, goats, and cattle or vice versa can occur, a constant concern for both wildlife management and agricultural production (Gortazar et al., 2015; Beaumelle et al., 2022). More than 2 million sheep graze in alpine areas of Norway every summer in habitat shared with endemic ungulates. We detected multiple nematode taxa in moose that are more typical parasites of domestic animals such as *Chabertia ovina*, *H*. *contortus*, *O*. *venulosum*, *Teldorsagia circumcincta*, *Trichostrongylus colubriformis*, *T. axis*, and *Trichuris ovis*, all of which were found primarily in central and southern Norway where sheep and cattle densities are highest (Table 1, Table 2). These finds strongly suggest the spillover of parasites from domestic animals to moose, although the generally low prevalence of these parasites suggests that moose are a poor reservoir for spillback into domestic animals. Several of these nematodes are highly pathogenic in sheep, goats, and cattle such as *H. contortus* and *T. circumcincta* causing anemia and premature death leading to large economic losses (Hunter and Mackenzie, 1982; Lane et al., 2015). It is currently unknown if they induce similar pathogenesis in moose, but notably, one calf in Trøndelag was found deceased one month post collaring and tested positive for *Nematodirus battus* at time of death (unpublished). *Nematodirus battus* is another major pathogenic nematode of sheep causing severe disease especially in young lambs (Thomas, 1991). It is uncertain to what extent this nematode had a role in the mortality of the individual. Regardless our findings suggest a potential risk of spillover of multiple pathogenic nematodes from domestic animals to moose in central and southern Norway that may have important implications for wildlife management.

### 4.2 Climate and seasonality

Climatic variables such as temperature and precipitation directly impact the survival and growth rate of the free-living stage of parasitic nematodes. At 10 °C or below both the hatching of eggs and development to infective third stage larva can take weeks to months as opposed to hours or days at higher temperatures (Ciordia and Bizzel, 1963; Crofton, 1965). Furthermore, eggs generally will not successfully hatch below 5 °C (Ciordia and Bizzel, 1963; Crofton, 1965). Indeed, in Hardangervidda where snow covers the ground for all but a few months each year and temperatures in summer are mild, the least number of nematode taxa were detected (Fig. 2, S1, Table 2). Those identified are likely moose specialists such as *Nematodirella alcidis* (Hoberg et al., 2001). Although the genus *Nematodirella* was detected, the presence of *N. alcidis* specifically could not be confirmed due to a lack of reference sequences. The period for nematodes to reach their freeze resistant infective stage may not be long enough for generalist species in some of the study areas. Notably, *H. contortus* was not detected in either Finnmark or Hardangervidda despite the presence of grazing sheep. Our samples from moose in Finnmark are further north (70°N) than the northernmost record in domestic sheep in Norway (68°N; Domke et al., 2013). The eggs of this nematode do not survive freezing and require temperatures above 16 °C to hatch which may not be reached in these localities even in summer (Ransom, 1906; Gordon, 1948). Therefore, the risk of spillover to moose may currently be exceedingly low in these areas but could be expected to increase as the climate warms.

Surprisingly, we found lower nematode richness and prevalence in summer with no nematode DNA being recovered from over half of the individuals, although only females were sampled, and the relatively low sample size may limit accurate prevalence estimates of rare nematode taxa (Table 3). Nevertheless, this observation is in stark contrast to what is typically reported in large mammals in northern latitudes, where fecal egg counts peak in spring and summer when conditions are ideal for the development and transmission of larva (Stien et al., 2002; O'Connor et al., 2006; Wood et al., 2013; Albery et al., 2018). Eggs may not be as resilient to freezing during the harsh winters as third stage infective larva remaining in the environment from summer and autumn (Crofton, 1965). Despite this, there is tantalizing evidence of an alternate transmission pattern of nematodes regardless of the harsh winter experienced by these animals. Filip-Hutsch et al. (2020) reported higher prevalence of *Nematodirella alcidis* in moose feces in winter as compared to summer, similar to our own findings. Winter transmission of the generalist nematode *Marshallagia marshalli* has also been demonstrated experimentally in reindeer on the high arctic island of Svalbard (Carlsson et al., 2012). Further, adult worms have been recovered from the intestines of red deer and reindeer (Hrabok et al., 2009; Davidson et al., 2014) as well as eggs from moose feces in winter (Milner et al., 2013). These observations suggest winter may be the primary season for nematode egg production in moose in Norway despite conditions appearing to be less than ideal.

Many ruminant nematodes including those detected here are able to arrest their development within the host (i.e. hypobiosis) until specific conditions such as a reduced immune response are met for successful development into egg producing adults (Michel, 1974). Moose have adapted to the lower quality diet in winter by reducing their metabolism and undergoing varying degrees of weight loss (Regelin et al., 1985; Schwartz et al., 1987), thereby resulting in lowered body condition that may be associated with higher parasite load (Davidson et al., 2015). The lowered body condition may reduce the effectiveness of the immune system of moose triggering larva to leave hypobiosis when the host may not be able to fight off infection. On the other hand, many of the nematode taxa we detected are not moose specific but can infect other wild ungulates or livestock and are unlikely to have adapted specifically to moose ecology. Future studies should compare the seasonal differences in nematode communities among multiple wild ungulate species in the subarctic and arctic to determine if winter egg production is a common trend in wild animals or if the results reported here are an exception.

### 4.3 Habitat use & migration

Habitat use affects home range overlap with other individuals and host species, thereby affecting transmission of parasites and pathogens (Dougherty et al., 2018; Mysterud et al., 2023). Habitat overlap among cervids varies seasonally, and depending on migratory behavior can involve a shift in seasonal home range placement (Mysterud et al., 2023). Variation in climate such as temperature, moisture, and snow cover among habitat types can also affect the survival of nematode eggs and larva (Ransom, 1906; Ciordia and Bizzel, 1963; Crofton, 1965). In this study, within home range habitat use patterns had no effect on nematode richness (except the use of grazing pastures in the global model), beta-diversity, or prevalence for the five nematodes analyzed. The limited role of habitat use may be due to the majority of time being spent within forested areas (Fig. S3).

The relationship between migratory behavior and parasitism remains relatively understudied, and both increased and reduced levels of parasitism have been reported (Folstad et al., 1991; Normandeau et al., 2020; Binning et al., 2022). We found the effect of migration distance on nematode taxa richness was not consistent across all study areas but was associated with a differential nematode community (i.e. beta-diversity). At both Gausdal-Murudal and Trøndelag, the further individuals migrated the higher the nematode richness they harbored. At Gausdal-Murudal where migration distances were longest, this appears to be driven by a higher risk of infection with *Nematodirella* spp. and *T. axei*. Individuals that travel further likely pass through a higher number of home ranges of other ungulate individuals as well as sheep and cattle pastures, thereby increasing the chance of encounters with these nematodes. In Trøndelag where migration distance is shortest, the increased nematode taxa richness was not driven by a single parasite. Because moose densities in this area are highest (based on hunting data) migratory individuals may still pass through a sufficient number of other home ranges to acquire new nematodes while not traveling far enough to encounter those species not found locally. In contrast, migration distance was associated with higher risk of infection with *E. alces* and elevation change with *Trichostrongylus* spp. at Valdres-Halldingdal, thereby affecting the parasitic nematode communities they harbor but not overall richness. Our findings suggest that infection patterns associated with host migration is a byproduct of such behavior and that it highly depends upon local context.

### 4.4 Age and sex

The higher nematode richness as well as higher prevalence of *E. alces* and *Nematodirella* spp. in calves than adults is consistent with a commonly reported trend in ruminants, usually attributed to the effect of acquired immunity (Hayward et al., 2011; Benton et al., 2018). However, the effect of age on the probability of infection of nematodes in moose is found to vary (Davidson et al., 2015) and should be subject to further investigation.

There was no clear effect of sex on infection patterns as generally would be expected (Benton et al., 2018; Świslocka et al., 2020). Previous studies of moose in Europe have found varying effects of sex on infection patterns of parasites (Davidson et al., 2015; Świslocka et al., 2020; Filip-Hutsch et al., 2021). These varying effects may be linked to other factors such as season, host state (e.g. pregnancy) or behavior playing a larger role in shaping the nematode communities infecting these animals.

### 4.5 Limitations of metabarcoding

The application of molecular methods for characterizing the parasite community of wildlife using feces has been gaining interest (Świslocka et al., 2020; Beaumelle et al., 2021; Davey et al., 2023). This non-invasive method allows for higher taxonomic resolution than morphological identification of eggs and larva and can utilize samples stored over long periods of time (Greiman et al., 2018; Davey et al., 2023). However, the lack of sufficient reference sequences available in public repositories remains a major obstacle that prevented the identification of multiple nematode taxa to species level in this study, thereby possibly affecting richness estimates. Furthermore, metabarcoding is a semi-quantitative method of fecal egg and larva counts that are themselves not always a reliable proxy for infection intensity (Eysker and Ploeger, 2000; Davidson et al., 2015). Despite these limitations, our study has demonstrated that DNA metabarcoding is a powerful tool for characterizing the nematode community of wildlife and for detecting spillover events across broader scales and time spans as compared to traditional, more labor-intensive methods.

## 5 Conclusion

Using DNA metabarcoding we were able to characterize the parasitic nematode community of moose over a large latitudinal gradient and explore potential drivers of infection patterns. The lack of a latitudinal gradient indicates that local context is an important driver of parasite community structure. We found evidence of spillover from domestic sheep or cattle to moose, and population-specific effects of migration on parasite infections. These population-specific effects in moose may imply that parasite infection patterns result as a bi-product of migration rather than a driving force behind its evolution (Bauer and Hoye, 2014). Moose are a keystone species with a wide distribution across the northern hemisphere, and they already face challenges with increased parasitic infections under climate change at their southern distribution range in North America (Murray et al., 2006; Debow et al., 2021). Our study provides a baseline for further studies into the parasite community in moose across a wider geographic area and highlights the role of local details in habitat sharing, land use, and migratory behavior in shaping the parasite community relevant for moose and other northern cervids.

## Funding

We are grateful to the 10.13039/501100008776Norwegian Environment Agency for funding the projects that have captured and marked moose in Trøndelag and Hardangervidda. The projects in Gausdal-Murudal, Valdres-Hallingdal and Finnmark were funded by multiple sources including municipalities, counties, landowners, the 10.13039/501100008776Norwegian Environment Agency and Regional research fund Nord (Finnmark). The research was also supported by the basic funding of the 10.13039/501100019715Norwegian Institute for Nature Research, financed by The Research Council of Norway (project no. 160022/F40).

## Declarations of interest

None

## CRediT authorship contribution statement

**Jason L. Anders:** Writing – review & editing, Writing – original draft, Visualization, Project administration, Methodology, Formal analysis, Conceptualization. **Marie Davey:** Writing – review & editing, Methodology, Formal analysis. **Bram Van Moorter:** Writing – review & editing, Formal analysis. **Frode Fossøy:** Writing – review & editing, Project administration, Methodology. **Sanne Boessenkool:** Writing – review & editing, Methodology, Conceptualization. **Erling J. Solberg:** Writing – review & editing, Investigation, Data curation. **Erling L. Meisingset:** Writing – review & editing, Investigation, Data curation. **Atle Mysterud:** Writing – review & editing, Project administration, Conceptualization. **Christer M. Rolandsen:** Writing – review & editing, Project administration, Investigation, Funding acquisition, Formal analysis, Data curation, Conceptualization.

## Declaration of competing interest

None.
