# Supplementary material for: Elucidating nematode diversity and prevalence in moose across a wide latitudinal gradient using DNA metabarcoding
Source: Int J Parasitol Parasites Wildl. 2024 Jul 5;24:100962. doi: 10.1016/j.ijppaw.2024.100962 (PMC11295938; doi:10.1016/j.ijppaw.2024.100962)
Supplement: Multimedia component 1 [file mmc1.docx]

**Supplemental Information**

**Additional materials and methods**

*Study area description*

Moose may spend substantial time above the woodland limit (about 1500 m.a.s.l.) during summer, while at Hardangervidda most moose stay at or above the woodland limit all year round. The elevation of summer and winter home ranges varied greatly among study areas with the highest elevations in Hardangervidda and the lowest in Finnmark (Table 1).

**Additional results**

*Habitat use patterns*

Average proportional use of different habitat types varied among study areas with the greatest variation being time spent in deciduous or coniferous forests (Fig. S3). Average proportional time spent in coniferous forests ranged from 0% in Hardangervidda to 76.2% in Trøndelag while individuals in Finnmark spent 66.6% of their time in deciduous forests on average as compared to Trøndelag with only 2.7% (Fig. S3).

*Migration behavior*

The longest migration distances were observed in Gausdal-Murudal while the shortest were in Trøndelag (Table 1). The summer home range in Gausdal-Murudal was at a lower elevation than the winter home range for more than half (24 of 41) of the individuals. A similar trend was found for 16 of 60 individuals in Trøndelag and 7 of 17 individuals in Hardangervidda, although the elevational decrease was not as large as in Gausdal-Murudal (Table 1).

**The Number of OTUs per Nematode Taxa**

| **Nematode Taxa** | **# of OTUs** |
| --- | --- |
| *Bunostomum* sp. | 1 |
| ***Chabertia ovina*** | 1 |
| *Chabertia* sp. | 1 |
| *Cooperia* spp. | 6 |
| *Elaphostrongylus alces* | 2 |
| *Elaphostrongylus* spp. | 3 |
| ***Haemonchus contortus*** | 3 |
| *Nematodirella* spp. | 69 |
| ***Oesophagostomum venulosum*** | 2 |
| *Ostertagia gruehneri* | 1 |
| *Ostertagia ostertagi* | 2 |
| *Ostertagia* spp. | 22 |
| *Spiculopteragia boehmi* | 2 |
| *Spiculopteragia* spp. | 4 |
| ***Teladorsagia circumcincta*** | 4 |
| ***Trichostrongylus axei*** | 1 |
| ***Trichostrongylus colubriformis*** | 5 |
| *Trichostrongylus* spp. | 14 |
| ***Trichuris ovis*** | 1 |
| Unclassified 1 (Haemonchidae) | 1 |
| Unclassified 2 (Strongylida) | 1 |
| **Total** | 146 |

**Table S1:** The number of OTUs included in each nematode taxa after collapsing to the lowest taxonomic level. Species in bold also occur in domestic sheep and goats (Domke et al. 2013).

**Geographic nematode richness (global model)**

|  | **b** | **SE** | **t-value** | ***p*** |
| --- | --- | --- | --- | --- |
| (Intercept) | 3.144 | 0.377 | 8.337 | **< 0.001** |
| Sex (Male) | 0.132 | 0.178 | 0.738 | 0.462 |
| Age Class (Calf) | 0.732 | 0.373 | 1.962 | 0.052 |
| Age Class (Unknown) | -0.067 | 0.350 | -0.192 | 0.848 |
| Age Class (Yearling) | 0.384 | 0.337 | 1.141 | 0.255 |
| Study Area (Gausdal-Murudal) | 0.004 | 0.440 | 0.010 | 0.992 |
| Study Area (Hardangervidda) | -1.037 | 0.506 | -2.049 | **0.045** |
| Study Area (Trøndelag) | -0.142 | 0.269 | -0.526 | 0.599 |
| Study Area (Valdres-Hallingdal) | -0.475 | 0.629 | -0.755 | 0.489 |

**Table S2:** LMM results for the global geographic model comparing nematode taxa richness across all study areas, host age class, and host sex. Year is the random variable. The base value for the categorical variable sex is female, for age-class is adult, and study area is Finnmark.

**Nematode richness geographic model Tukey’s post-hoc analysis**

| **Study Area** |  |  |  |  |
| --- | --- | --- | --- | --- |
|  | **b** | **SE** | **z-value** | ***p*** |
| Gausdal-Murudal - Finnmark | 0.004 | 0.440 | 0.01 | 1.000 |
| Hardangervidda - Finnmark | -1.037 | 0.506 | -2.049 | 0.220 |
| Trøndelag - Finnmark | -0.142 | 0.269 | -0.526 | 0.983 |
| Valdres-Hallingdal - Finnmark | -0.475 | 0.629 | -0.755 | 0.936 |
| Hardangervidda - Gausdal-Murudal | -1.041 | 0.350 | -2.976 | **0.021** |
| Trøndelag - Gausdal-Murudal | -0.146 | 0.386 | -0.379 | 0.995 |
| Valdres-Hallingdal – Gausdal-Murudal | -0.480 | 0.670 | -0.716 | 0.947 |
| Trøndelag - Hardangervidda | 0.895 | 0.465 | 1.926 | 0.278 |
| Valdres-Hallingdal - Hardangervidda | 0.562 | 0.715 | 0.786 | 0.927 |
| Valdres-Hallingdal - Trøndelag | -0.334 | 0.634 | -0.526 | 0.983 |
|  |  |  |  |  |
| **Age Class** |  |  |  |  |
|  | **b** | **SE** | **z-value** | ***p*** |
| Calf - Adult | 0.732 | 0.373 | 1.962 | 0.191 |
| Unknown - Adult | -0.067 | 0.350 | -0.192 | 0.997 |
| Yearling - Adult | 0.384 | 0.337 | 1.141 | 0.649 |
| Unknown - Calf | -0.800 | 0.485 | -1.65 | 0.335 |
| Yearling - Calf | -0.348 | 0.478 | -0.728 | 0.879 |
| Yearling - Unknown | 0.452 | 0.477 | 0.948 | 0.767 |

**Table S3:** Tukey’s post-hoc analyses for the global geographic model comparing among study area and among age class differences in nematode taxa richness.

**Geographic Model Beta-Diversity**

|  | **SS** | **R^2^** | **F** | ***p*** |
| --- | --- | --- | --- | --- |
| Sex | 0.120 | 0.004 | 1.178 | 0.323 |
| Age Class | 0.843 | 0.031 | 2.759 | **0.001** |
| Year | 0.164 | 0.006 | 1.613 | 0.160 |
| Study Area | 2.507 | 0.093 | 6.153 | **0.001** |

**Table S4:** PERMANOVA results for the global geographic model with Jaccard dissimilarity comparing nematode community structure across study area, host age class, host sex, and sample year.

**Geographic nematode taxa prevalence (global model)**

|  | ***Nematodirella spp.*** | | | | ***Elaphostrongylus alces*** | | | | ***Trichostrongylus axei*** | | | |
| --- | --- | --- | --- | --- | --- | --- | --- | --- | --- | --- | --- | --- |
|  | **b** | **SE** | **z-value** | ***p*** | **b** | **SE** | **z-value** | ***p*** | **b** | **SE** | **z-value** | ***p*** |
| (Intercept) | 0.200 | 0.311 | 0.643 | 0.520 | **−** | **−** | **−** | **−** | -2.741 | 0.637 | -4.306 | **< 0.001** |
| Sex (male) | -0.232 | 0.313 | -0.742 | 0.458 | **−** | **−** | **−** | **−** | 0.205 | 0.465 | 0.440 | 0.660 |
| Age class (Calf) | 1.895 | 0.698 | 2.715 | **0.007** | **−** | **−** | **−** | **−** | -0.382 | 0.870 | -0.439 | 0.660 |
| Age class (Unknown) | 0.027 | 0.481 | 0.057 | 0.955 | **−** | **−** | **−** | **−** | -0.479 | 1.099 | -0.436 | 0.663 |
| Age class (Yearling) | 0.565 | 0.593 | 0.952 | 0.341 | **−** | **−** | **−** | **−** | -1.030 | 1.094 | -0.941 | 0.347 |
| Study area (Gausdal-Murudal) | 0.396 | 0.438 | 0.905 | 0.366 | **−** | **−** | **−** | **−** | 1.768 | 0.715 | 2.472 | **0.013** |
| Study area (Hardangervidda) | 1.599 | 0.698 | 2.291 | **0.022** | **−** | **−** | **−** | **−** | 15.893 | 1455.519 | -0.011 | 0.991 |
| Study area (Trøndelag) | -0.246 | 0.388 | -0.634 | 0.526 | **−** | **−** | **−** | **−** | 0.840 | 0.721 | 1.164 | 0.245 |
| Study area (Valdres-Hallingdal) | -0.013 | 0.438 | -0.030 | 0.976 | **−** | **−** | **−** | **−** | -0.020 | 0.945 | -0.021 | 0.983 |
|  | ***Trichostongylus spp.*** | | | | ***Unclassified Strongylida*** | | | |  |  |  |  |
|  | **b** | **SE** | **z-value** | ***p*** | **b** | **SE** | **z-value** | ***p*** |  |  |  |  |
| (Intercept) | -2.552 | 0.618 | -4.127 | **< 0.001** | 1.609 | 0.410 | 3.928 | **< 0.001** |  |  |  |  |
| Sex (male) | -0.883 | 0.475 | -1.859 | 0.063 | 0.111 | 0.341 | 0.326 | 0.744 |  |  |  |  |
| Age class (Calf) | -1.344 | 0.820 | -1.638 | 0.101 | -0.731 | 0.559 | -1.308 | 0.191 |  |  |  |  |
| Age class (Unknown) | -0.305 | 0.837 | -0.365 | 0.715 | 0.429 | 0.596 | 0.720 | 0.471 |  |  |  |  |
| Age class (Yearling) | -0.350 | 0.841 | -0.416 | 0.677 | -0.845 | 0.598 | -1.412 | 0.158 |  |  |  |  |
| Study area (Gausdal-Murudal) | 1.382 | 0.736 | 1.877 | 0.061 | -0.787 | 0.520 | -1.512 | 0.131 |  |  |  |  |
| Study area (Hardangervidda) | -14.574 | 861.963 | -0.017 | 0.987 | -3.859 | 0.847 | -4.558 | **< 0.001** |  |  |  |  |
| Study area (Trøndelag) | 2.290 | 0.663 | 3.453 | **< 0.001** | -0.860 | 0.480 | -1.791 | 0.073 |  |  |  |  |
| Study area (Valdres-Hallingdal) | 0.690 | 0.803 | 0.859 | 0.390 | -2.133 | 0.517 | -4.125 | **< 0.001** |  |  |  |  |

**Table S5:** GLM binomial model results comparing nematode taxa prevalence across all study areas (i.e. global model), host age, class, and host sex. The base value for the categorical variable sex is female, for age-class is adult, and study area is Finnmark.

**Geographic nematode taxa prevalence Tukey’s posthoc analyses (study area)**

|  | ***Nematodirella spp.*** | | | | ***Elaphostrongylus alces*** | | | | ***Trichostrongylus axei*** | | | |
| --- | --- | --- | --- | --- | --- | --- | --- | --- | --- | --- | --- | --- |
|  | **b** | **SE** | **z-value** | ***p*** | **b** | **SE** | **z-value** | ***p*** | **b** | **SE** | **z-value** | ***p*** |
| Gausdal-Murudal - Finnmark | 0.396 | 0.438 | 0.905 | 0.890 | **−** | **−** | **−** | **−** | 1.768 | 0.715 | 2.472 | 0.074 |
| Hardangervidda - Finnmark | 1.599 | 0.698 | 2.291 | 0.140 | **−** | **−** | **−** | **−** | -15.893 | 1455.519 | -0.011 | 1.000 |
| Trøndelag - Finnmark | -0.246 | 0.388 | -0.634 | 0.968 | **−** | **−** | **−** | **−** | 0.84 | 0.721 | 1.164 | 0.729 |
| Valdres-Hallingdal - Finnmark | -0.013 | 0.438 | -0.030 | 1.000 | **−** | **−** | **−** | **−** | -0.02 | 0.945 | -0.021 | 1.000 |
| Hardangervidda - Gausdal-Murudal | 1.203 | 0.707 | 1.703 | 0.420 | **−** | **−** | **−** | **−** | -17.661 | 1455.519 | -0.012 | 1.000 |
| Trøndelag - Gausdal-Murudal | -0.643 | 0.411 | -1.563 | 0.509 | **−** | **−** | **−** | **−** | -0.928 | 0.52 | -1.784 | 0.327 |
| Valdres-Hallingdal - Gausdal-Murudal | -0.409 | 0.465 | -0.881 | 0.900 | **−** | **−** | **−** | **−** | -1.788 | 0.818 | -2.185 | 0.148 |
| Trøndelag - Hardangervidda | -1.846 | 0.685 | -2.694 | 0.052 | **−** | **−** | **−** | **−** | 16.733 | 1455.519 | 0.011 | 1.000 |
| Valdres-Hallingdal - Hardangervidda | -1.612 | 0.722 | -2.233 | 0.160 | **−** | **−** | **−** | **−** | 15.873 | 1455.519 | 0.011 | 1.000 |
| Valdres-Hallingdal - Trøndelag | 0.233 | 0.423 | 0.551 | 0.981 | **−** | **−** | **−** | **−** | -0.86 | 0.829 | -1.037 | 0.804 |
|  | ***Trichostongylus spp.*** | | | | **Unclassified 2 (Strongylida)** | | | |  |  |  |  |
|  | **b** | **SE** | **z-value** | ***p*** | **b** | **SE** | **z-value** | ***p*** |  |  |  |  |
| Gausdal-Murudal – Finnmark | 1.382 | 0.736 | 1.877 | 0.277 | -0.787 | 0.52 | -1.512 | 0.541 |  |  |  |  |
| Hardangervidda – Finnmark | -14.574 | 861.963 | -0.017 | 1.000 | -3.859 | 0.847 | -4.558 | **< 0.001** |  |  |  |  |
| Trøndelag – Finnmark | 2.29 | 0.663 | 3.453 | **0.004** | -0.86 | 0.48 | -1.791 | 0.365 |  |  |  |  |
| Valdres-Hallingdal – Finnmark | 0.69 | 0.803 | 0.859 | 0.89 | -2.133 | 0.517 | -4.125 | **< 0.001** |  |  |  |  |
| Hardangervidda – Gausdal-Murudal | -15.956 | 861.962 | -0.019 | 1.000 | -3.073 | 0.82 | -3.747 | **0.002** |  |  |  |  |
| Trøndelag – Gausdal-Murudal | 0.908 | 0.481 | 1.887 | 0.272 | -0.073 | 0.433 | -0.169 | 1.000 |  |  |  |  |
| Valdres-Hallingdal – Gausdal-Murudal | -0.692 | 0.677 | -1.022 | 0.812 | -1.346 | 0.485 | -2.776 | **0.041** |  |  |  |  |
| Trøndelag – Hardangervidda | 16.864 | 861.962 | 0.02 | 1.000 | 2.999 | 0.799 | 3.751 | **0.001** |  |  |  |  |
| Valdres-Hallingdal – Hardangervidda | 15.264 | 861.963 | 0.018 | 1.000 | 1.726 | 0.831 | 2.078 | 0.218 |  |  |  |  |
| Valdres-Hallingdal – Trøndelag | -1.6 | 0.601 | -2.661 | **0.045** | -1.273 | 0.446 | -2.854 | **0.032** |  |  |  |  |

**Table S6:** Tukey’s post-hoc analyses for study area in the global geographic nematode prevalence GLMs.

**Geographic nematode taxa prevalence Tukey’s posthoc analyses (age class)**

|  | ***Nematodirella spp.*** | | | | ***Elaphostrongylus alces*** | | | | ***Trichostrongylus axei*** | | | |
| --- | --- | --- | --- | --- | --- | --- | --- | --- | --- | --- | --- | --- |
|  | **b** | **SE** | **z-value** | ***p*** | **b** | **SE** | **z-value** | ***p*** | **b** | **SE** | **z-value** | ***p*** |
| Calf – Adult | 1.346 | 0.604 | 2.23 | 0.105 | **−** | **−** | **−** | **−** | -0.382 | 0.87 | -0.439 | 0.969 |
| Unknown – Adult | -0.186 | 0.623 | -0.298 | 0.99 | **−** | **−** | **−** | **−** | -0.479 | 1.099 | -0.436 | 0.97 |
| Yearling – Adult | -0.859 | 1.077 | -0.798 | 0.844 | **−** | **−** | **−** | **−** | -1.03 | 1.094 | -0.941 | 0.768 |
| Unknown – Calf | -1.532 | 0.841 | -1.82 | 0.245 | **−** | **−** | **−** | **−** | -0.097 | 1.364 | -0.071 | 1.000 |
| Yearling – Calf | -2.205 | 1.19 | -1.853 | 0.23 | **−** | **−** | **−** | **−** | -0.648 | 1.361 | -0.476 | 0.961 |
| Yearling – Unknown | -0.674 | 1.213 | -0.555 | 0.94 | **−** | **−** | **−** | **−** | -0.551 | 1.522 | -0.362 | 0.982 |
|  | ***Trichostongylus spp.*** | | | | **Unclassified 2 (Strongylida)** | | | |  |  |  |  |
|  | **b** | **SE** | **z-value** | ***p*** | **b** | **SE** | **z-value** | ***p*** |  |  |  |  |
| Calf – Adult | -1.344 | 0.82 | -1.638 | 0.34 | -0.731 | 0.559 | -1.308 | 0.541 |  |  |  |  |
| Unknown – Adult | -0.305 | 0.837 | -0.365 | 0.982 | 0.429 | 0.596 | 0.72 | 0.882 |  |  |  |  |
| Yearling – Adult | -0.35 | 0.841 | -0.416 | 0.974 | -0.845 | 0.598 | -1.412 | 0.475 |  |  |  |  |
| Unknown – Calf | 1.038 | 1.142 | 0.91 | 0.787 | 1.161 | 0.798 | 1.455 | 0.448 |  |  |  |  |
| Yearling – Calf | 0.993 | 1.136 | 0.874 | 0.806 | -0.114 | 0.772 | -0.147 | 0.999 |  |  |  |  |
| Yearling – Unknown | -0.045 | 1.154 | -0.039 | 1.000 | -1.274 | 0.808 | -1.576 | 0.376 |  |  |  |  |

**Table S7:** Tukey’s post-hoc analyses for host age class in the global geographic nematode prevalence GLMs.

**Habitat use and nematode taxa richness (global model)**

|  | **b** | **SE** | **t-value** | ***p*** |
| --- | --- | --- | --- | --- |
| (Intercept) | 2.757 | 0.362 | 7.606 | **< 0.001** |
| Sex (male) | 0.259 | 0.190 | 1.359 | 0.176 |
| Age class (Calf) | 1.047 | 0.380 | 2.752 | **0.007** |
| Age class (Unknown) | 0.388 | 0.385 | 1.007 | 0.316 |
| Age class (Yearling) | 0.345 | 0.350 | 0.986 | 0.326 |
| prop. deciduous forest | 0.012 | 0.444 | 0.027 | 0.979 |
| prop. mixed forest | -1.930 | 1.336 | -1.444 | 0.151 |
| prop. agriculture | 0.444 | 1.400 | 0.317 | 0.752 |
| prop. marshland | 2.507 | 1.943 | 1.290 | 0.199 |
| prop. open land | -1.527 | 1.039 | -1.470 | 0.143 |
| prop. grazing pasture | 14.404 | 7.188 | 2.004 | **0.047** |

**Table S8:** LMM results for the global habitat use model exploring the proportional use of different habitat types on nematode taxa richness across all study areas (i.e. global model). Year and study area are the random variables. The base value for the categorical variable sex is female and for age-class is adult.

**Habitat use and nematode taxa beta-diversity (global model)**

|  | **SS** | **R^2^** | **F** | ***p*** |
| --- | --- | --- | --- | --- |
| Year | 0.135 | 0.004 | 0.866 | 0.512 |
| Sex | 0.199 | 0.006 | 1.282 | 0.250 |
| Age Class | 0.979 | 0.029 | 2.098 | **0.003** |
| Study Area | 2.765 | 0.082 | 4.444 | **0.001** |
| Prop. deciduous forest | 0.133 | 0.004 | 0.858 | 0.511 |
| Prop. mixed forest | 0.315 | 0.009 | 2.027 | 0.064 |
| Prop. agriculture | 0.034 | 0.001 | 0.217 | 0.944 |
| Prop. marshland | 0.108 | 0.003 | 0.695 | 0.640 |
| Prop. open land | 0.107 | 0.003 | 0.688 | 0.650 |
| Prop. grazing pasture | 0.175 | 0.005 | 1.127 | 0.346 |

**Table S9:** PERMANOVA results for the global habitat use model testing the proportional use of different habitat types on nematode taxa beta-diversity across all study areas (i.e. global model) based on Jaccard dissimilarity.

**Habitat use and nematode taxa richness (study area specific)**

|  | **Finnmark (LM)** | | | | **Trondelag (LM)** | | | |
| --- | --- | --- | --- | --- | --- | --- | --- | --- |
|  | **b** | **SE** | **t-value** | ***p*** | **b** | **SE** | **t-value** | ***p*** |
| (Intercept) | 1.598 | 0.822 | 1.944 | 0.060 | 2.856 | 0.370 | 7.711 | **< 0.001** |
| Sex (male) | 0.375 | 0.378 | 0.991 | 0.328 | 0.391 | 0.354 | 1.104 | 0.275 |
| Age class (Calf) | **−** | **−** | **−** | **−** | 1.272 | 0.413 | 3.082 | **0.003** |
| Age class (Unknown) | 0.139 | 0.501 | 0.278 | 0.783 | -0.005 | 0.907 | -0.006 | 0.995 |
| Age class (Yearling) | 0.741 | 1.095 | 0.676 | 0.503 | 2.408 | 0.740 | 3.252 | **0.002** |
| prop. deciduous forest | 1.885 | 0.946 | 1.992 | 0.055 | **−** | **−** | **−** | **−** |
| prop. mixed forest | **−** | **−** | **−** | **−** | -5.400 | 5.790 | -0.933 | 0.355 |
| prop. agriculture | **−** | **−** | **−** | **−** | **−** | **−** | **−** | **−** |
| prop. marshland | -2.254 | 2.592 | -0.870 | 0.391 | 3.755 | 3.339 | 1.125 | 0.266 |
| prop. open land | 0.975 | 2.082 | 0.468 | 0.643 | -1.540 | 2.433 | -0.633 | 0.529 |
|  | **Gausdal-Murudal (LM)** | | | | **Valdres-Hallingdal (LM)** | | | |
|  | **b** | **SE** | **t-value** | ***p*** | **b** | **SE** | **t-value** | ***p*** |
| (Intercept) | 4.085 | 0.788 | 5.185 | **< 0.001** | 2.126 | 0.314 | 6.771 | **< 0.001** |
| Sex (male) | 0.787 | 0.522 | 1.508 | 0.141 | -0.135 | 0.366 | -0.370 | 0.714 |
| Age class (Calf) | **−** | **−** | **−** | **−** | **−** | **−** | **−** | **−** |
| Age class (Unknown) | **−** | **−** | **−** | **−** | 1.968 | 0.405 | 4.862 | **< 0.001** |
| Age class (Yearling) | -1.044 | 0.845 | -1.235 | 0.225 | 0.870 | 0.460 | 1.892 | 0.070 |
| prop. deciduous forest | -2.241 | 1.858 | -1.206 | 0.236 | -0.152 | 0.819 | -0.186 | 0.854 |
| prop. mixed forest | -2.453 | 2.996 | -0.819 | 0.418 | -0.952 | 1.352 | -0.705 | 0.488 |
| prop. agriculture | **−** | **−** | **−** | **−** | **−** | **−** | **−** | **−** |
| prop. marshland | -1.692 | 6.828 | -0.248 | 0.806 | -7.109 | 6.112 | -1.163 | 0.256 |
| prop. open land | -0.642 | 3.943 | -0.163 | 0.872 | 2.382 | 2.427 | 0.982 | 0.336 |
|  | **Hardangervidda (LM)** | | | |  |  |  |  |
|  | **b** | **SE** | **t-value** | ***p*** |  |  |  |  |
| (Intercept) | 2.265 | 0.405 | 5.599 | **< 0.001** |  |  |  |  |
| Sex (male) | -0.240 | 0.524 | -0.458 | 0.658 |  |  |  |  |
| Age class (Calf) | -0.488 | 1.059 | -0.461 | 0.656 |  |  |  |  |
| Age class (Unknown) | -0.265 | 0.914 | -0.290 | 0.778 |  |  |  |  |
| Age class (Yearling) | **−** | **−** | **−** | **−** |  |  |  |  |
| prop. deciduous forest | -0.189 | 0.970 | -0.195 | 0.850 |  |  |  |  |
| prop. mixed forest | **−** | **−** | **−** | **−** |  |  |  |  |
| prop. agriculture | -0.031 | 1.144 | -0.027 | 0.979 |  |  |  |  |
| prop. marshland | **−** | **−** | **−** | **−** |  |  |  |  |
| prop. open land | 1.322 | 2.576 | 0.513 | 0.620 |  |  |  |  |

**Table S10:** LM results exploring study area specific habitat use patterns on nematode taxa richness at each study area separately. Habitat types constituting less than 3% proportional use in a study area were excluded from the model. The base value for the categorical variable sex is female and for age-class is adult.

**Habitat use and nematode taxa beta-diversity (study area specific)**

|  | **Finnmark** | | | | **Trondelag** | | | |
| --- | --- | --- | --- | --- | --- | --- | --- | --- |
|  | **SS** | **R^2^** | **F** | ***p*** | **SS** | **R^2^** | **F** | ***p*** |
| Sex | 0.116 | 0.023 | 0.891 | 0.470 | 0.518 | 0.043 | 2.896 | **0.009** |
| Age Class | 0.240 | 0.047 | 0.926 | 0.466 | 1.243 | 0.102 | 2.315 | **0.002** |
| prop. deciduous forest | 0.176 | 0.035 | 1.361 | 0.249 | **−** | **−** | **−** | **−** |
| prop. mixed forest | **−** | **−** | **−** | **−** | 0.124 | 0.010 | 0.690 | 0.664 |
| prop. agriculture | **−** | **−** | **−** | **−** | **−** | **−** | **−** | **−** |
| prop. marshland | 0.025 | 0.005 | 0.190 | 0.953 | 0.315 | 0.026 | 1.762 | 0.093 |
| prop. open land | 0.048 | 0.009 | 0.370 | 0.850 | 0.127 | 0.010 | 0.711 | 0.649 |
|  | **Gausdal-Murudal** | | | | **Valdres-Hallingdal** | | | |
|  | **SS** | **R^2^** | **F** | ***p*** | **SS** | **R^2^** | **F** | ***p*** |
| Sex | 0.226 | 0.035 | 1.442 | 0.203 | 0.092 | 0.016 | 0.546 | 0.718 |
| Age Class | 0.189 | 0.029 | 1.208 | 0.305 | 0.803 | 0.143 | 2.376 | **0.020** |
| prop. deciduous forest | 0.198 | 0.030 | 1.263 | 0.287 | 0.230 | 0.041 | 1.358 | 0.239 |
| prop. mixed forest | 0.216 | 0.033 | 1.379 | 0.226 | 0.069 | 0.012 | 0.409 | 0.839 |
| prop. agriculture | **−** | **−** | **−** | **−** | **−** | **−** | **−** | **−** |
| prop. marshland | 0.206 | 0.031 | 1.311 | 0.263 | 0.142 | 0.025 | 0.841 | 0.477 |
| prop. open land | -0.010 | -0.001 | -0.062 | 0.994 | 0.076 | 0.014 | 0.448 | 0.795 |
|  | **Hardangervidda** | | | |  |  |  |  |
|  | **SS** | **R^2^** | **F** | ***p*** |  |  |  |  |
| Sex | 0.028 | 0.043 | 0.419 | 0.804 |  |  |  |  |
| Age Class | 0.008 | 0.013 | 0.062 | 0.933 |  |  |  |  |
| prop. deciduous forest | -0.008 | -0.012 | -0.120 | 0.981 |  |  |  |  |
| prop. mixed forest | **−** | **−** | **−** | **−** |  |  |  |  |
| prop. agriculture | 0.017 | 0.025 | 0.245 | 0.564 |  |  |  |  |
| prop. marshland | **−** | **−** | **−** | **−** |  |  |  |  |
| prop. open land | 0.018 | 0.027 | 0.261 | 0.682 |  |  |  |  |

**Table S11:** PERMANOVA results testing the effect of habitat use patterns on nematode taxa beta-diversity based on Jaccard dissimilarity. Habitat types constituting less than 3% proportional use in a study area were excluded from the model.

**Habitat use and nematode prevalence (global model)**

|  | ***Nematodirella spp.*** | | | | ***Elaphostrongylus alces*** | | | |
| --- | --- | --- | --- | --- | --- | --- | --- | --- |
|  | **coef** | **SE** | **Chisq** | ***p*** | **coef** | **SE** | **Chisq** | ***p*** |
| (Intercept) | -1.318 | 0.851 | 2.439 | 0.118 | -5.696 | 1.811 | 11.408 | **0.001** |
| Sex (male) | -0.163 | 0.327 | 0.245 | 0.621 | 1.047 | 0.49 | 4.102 | **0.043** |
| Age class (Calf) | 1.335 | 0.685 | 4.188 | **0.041** | 2.337 | 0.709 | 11.093 | **0.001** |
| Age class (Unknown) | 0.634 | 0.577 | 1.209 | 0.271 | 1.927 | 0.879 | 3.761 | 0.052 |
| Age class (Yearling) | 0.376 | 0.584 | 0.417 | 0.519 | 2.527 | 0.686 | 12.037 | **0.001** |
| Study area (Gausdal-Murudal) | 1.160 | 0.700 | 2.797 | 0.094 | 3.314 | 1.666 | 3.961 | **0.047** |
| Study area (Hardangervidda) | 2.931 | 1.020 | 11.052 | **0.001** | 3.126 | 1.655 | 3.209 | 0.073 |
| Study area (Trøndelag) | 0.823 | 0.800 | 1.056 | 0.304 | 3.141 | 1.791 | 2.836 | 0.092 |
| Study area (Valdres-Hallingdal) | 0.821 | 0.726 | 1.284 | 0.257 | 2.562 | 1.636 | 2.312 | 0.128 |
| Prop. deciduous forest | 1.458 | 1.032 | 2.017 | 0.156 | 0.408 | 1.792 | 0.04 | 0.842 |
| Prop. mixed forest | 1.445 | 2.313 | 0.388 | 0.533 | -3.013 | 4.348 | 0.415 | 0.52 |
| Prop. Agriculture | 1.099 | 3.019 | 0.115 | 0.735 | 0.402 | 2.342 | 0.027 | 0.869 |
| Prop. marshland | 3.321 | 3.296 | 1.016 | 0.313 | -2.495 | 4.941 | 0.235 | 0.628 |
| Prop. open land | 0.453 | 1.889 | 0.056 | 0.814 | -0.66 | 3.178 | 0.036 | 0.85 |
|  | ***Trichostrongylus axei*** | | | | ***Trichostongylus spp.*** | | | |
|  | **coef** | **SE** | **Chisq** | ***p*** | **coef** | **SE** | **Chisq** | ***p*** |
| (Intercept) | -0.588 | 1.173 | 0.217 | 0.641 | -2.811 | 1.244 | 4.804 | **0.028** |
| Sex (male) | 0.505 | 0.467 | 0.996 | 0.318 | -0.691 | 0.448 | 2.284 | 0.131 |
| Age class (Calf) | 0.14 | 0.85 | 0.025 | 0.874 | -0.729 | 0.764 | 0.949 | 0.33 |
| Age class (Unknown) | -0.168 | 0.922 | 0.029 | 0.865 | 0.237 | 0.789 | 0.08 | 0.777 |
| Age class (Yearling) | -0.77 | 0.967 | 0.599 | 0.439 | 0.093 | 0.754 | 0.014 | 0.906 |
| Study area (Gausdal-Murudal) | 1.122 | 1.051 | 0.965 | 0.326 | 1.886 | 1.047 | 2.842 | 0.092 |
| Study area (Hardangervidda) | -3.502 | 2.465 | 2.687 | 0.101 | -1.102 | 1.701 | 0.412 | 0.521 |
| Study area (Trøndelag) | -1.037 | 1.202 | 0.583 | 0.445 | 2.728 | 1.2 | 4.873 | **0.027** |
| Study area (Valdres-Hallingdal) | -0.628 | 1.179 | 0.229 | 0.632 | 0.96 | 1.102 | 0.621 | 0.431 |
| Prop. deciduous forest | -1.722 | 1.58 | 0.94 | 0.332 | 0.924 | 1.481 | 0.304 | 0.581 |
| Prop. mixed forest | -8.766 | 5.001 | 0.286 | 0.593 | -4.395 | 3.922 | 1.244 | 0.265 |
| Prop. Agriculture | 3.852 | 3.194 | 1.66 | 0.198 | 4.784 | 2.753 | 2.464 | 0.117 |
| Prop. marshland | 1.372 | 4.471 | 0.079 | 0.778 | -4.987 | 4.974 | 0.944 | 0.331 |
| Prop. open land | -7.947 | 4.32 | 3.311 | 0.069 | 2.15 | 2.428 | 0.639 | 0.424 |
|  | **Unclassified 2 (Strongylida)** | | | |  |  |  |  |
|  | **coef** | **SE** | **Chisq** | ***p*** |  |  |  |  |
| (Intercept) | 1.62 | 0.877 | 3.453 | 0.063 |  |  |  |  |
| Sex (male) | 0.039 | 0.348 | 0.012 | 0.913 |  |  |  |  |
| Age class (Calf) | -0.88 | 0.616 | 2.053 | 0.152 |  |  |  |  |
| Age class (Unknown) | 0.597 | 0.667 | 0.8 | 0.371 |  |  |  |  |
| Age class (Yearling) | -0.805 | 0.598 | 1.802 | 0.179 |  |  |  |  |
| Study area (Gausdal-Murudal) | -0.547 | 0.751 | 0.511 | 0.475 |  |  |  |  |
| Study area (Hardangervidda) | -3.161 | 0.885 | 16.079 | **<0.001** |  |  |  |  |
| Study area (Trøndelag) | -0.766 | 0.843 | 0.809 | 0.368 |  |  |  |  |
| Study area (Valdres-Hallingdal) | -1.858 | 0.779 | 5.956 | **0.015** |  |  |  |  |
| Prop. deciduous forest | 0.202 | 1.047 | 0.035 | 0.851 |  |  |  |  |
| Prop. mixed forest | -1.725 | 2.407 | 0.501 | 0.479 |  |  |  |  |
| Prop. Agriculture | 0.387 | 2.217 | 0.027 | 0.869 |  |  |  |  |
| Prop. marshland | -1.682 | 3.381 | 0.239 | 0.625 |  |  |  |  |
| Prop. open land | -1.086 | 1.935 | 0.299 | 0.585 |  |  |  |  |

**Table S12:** Results of Firth’s bias-reduced logistical regression testing the effect of habitat use patterns on the prevalence of six of the seven most common nematode taxa across all study areas (i.e. global model). The base value for the categorical variable sex is female, for age-class is adult, and for study area is Finnmark.

**Habitat use and nematode prevalence (study area specific)**

|  | ***Nematodirella spp.*** | | | | | | | |
| --- | --- | --- | --- | --- | --- | --- | --- | --- |
|  | **Finnmark** | | | | **Trondelag** | | | |
|  | **b** | **SE** | **Chisq** | ***p*** | **b** | **SE** | **Chisq** | ***p*** |
| (Intercept) | -1.621 | 1.665 | 1.111 | 0.292 | -0.649 | 0.712 | 0.766 | 0.382 |
| Sex (male) | 0.417 | 0.703 | 0.351 | 0.553 | -0.882 | 0.630 | 2.021 | 0.155 |
| Age class (Calf) | **−** | **−** | **−** | **−** | 1.883 | 0.803 | 6.457 | **0.011** |
| Age class (Unknown) | 1.017 | 0.991 | 1.136 | 0.286 | 0.568 | 1.251 | 0.202 | 0.653 |
| Age class (Yearling) | -1.250 | 1.777 | 0.552 | 0.457 | 1.714 | 1.549 | 1.635 | 0.201 |
| prop. deciduous forest | 1.742 | 1.887 | 0.954 | 0.329 | **−** | **−** | **−** | **−** |
| prop. mixed forest | **−** | **−** | **−** | **−** | -10.313 | 9.738 | 1.108 | 0.292 |
| prop. agriculture | **−** | **−** | **−** | **−** | **−** | **−** | **−** | **−** |
| prop. marshland | -0.287 | 4.700 | 0.004 | 0.952 | 10.539 | 7.350 | 2.027 | 0.155 |
| prop. open land | 2.336 | 3.609 | 0.413 | 0.521 | 3.406 | 4.008 | 0.712 | 0.399 |
|  | **Gausdal-Murudal** | | | | **Valdres-Hallingdal** | | | |
|  | **b** | **SE** | **Chisq** | ***p*** | **b** | **SE** | **Chisq** | ***p*** |
| (Intercept) | -0.680 | 0.975 | 0.486 | 0.486 | 0.210 | 0.800 | 0.068 | 0.794 |
| Sex (male) | 0.941 | 0.667 | 2.042 | 0.153 | -0.722 | 0.932 | 0.583 | 0.445 |
| Age class (Calf) | **−** | **−** | **−** | **−** | **−** | **−** | **−** | **−** |
| Age class (Unknown) | **−** | **−** | **−** | **−** | -0.693 | 1.031 | 0.451 | 0.502 |
| Age class (Yearling) | 0.009 | 0.987 | 0.000 | 0.993 | 0.537 | 1.151 | 0.213 | 0.644 |
| prop. deciduous forest | -1.618 | 2.153 | 0.570 | 0.450 | 3.111 | 2.232 | 2.081 | 0.149 |
| prop. mixed forest | 1.662 | 3.513 | 0.222 | 0.637 | 1.525 | 3.361 | 0.206 | 0.650 |
| prop. agriculture | **−** | **−** | **−** | **−** | **−** | **−** | **−** | **−** |
| prop. marshland | 11.572 | 8.669 | 1.863 | 0.172 | -15.485 | 16.323 | 0.932 | 0.334 |
| prop. open land | 1.934 | 4.888 | 0.154 | 0.695 | 0.867 | 6.361 | 0.018 | 0.894 |
|  | **Hardangervidda** | | | |  |  |  |  |
|  | **b** | **SE** | **Chisq** | ***p*** |  |  |  |  |
| (Intercept) | 1.967 | 1.351 | 2.869 | 0.090 |  |  |  |  |
| Sex (male) | -1.327 | 1.561 | 0.693 | 0.405 |  |  |  |  |
| Age class (Calf) | -0.223 | 2.220 | 0.010 | 0.921 |  |  |  |  |
| Age class (Unknown) | -0.869 | 2.120 | 0.156 | 0.693 |  |  |  |  |
| Age class (Yearling) | **−** | **−** | **−** | **−** |  |  |  |  |
| prop. deciduous forest | 1.189 | 3.191 | 0.119 | 0.730 |  |  |  |  |
| prop. mixed forest | **−** | **−** | **−** | **−** |  |  |  |  |
| prop. agriculture | 0.565 | 2.376 | 0.058 | 0.810 |  |  |  |  |
| prop. marshland | **−** | **−** | **−** | **−** |  |  |  |  |
| prop. open land | 0.234 | 6.258 | 0.001 | 0.972 |  |  |  |  |

**Table S13:** Results of Firth’s bias-reduced logistical regression testing the effect of habitat use patterns on the prevalence of six of the seven most common nematode taxa at each study area seperately. Habitat types constituting less than 3% proportional use in a study area were excluded from the model. The base value for the categorical variable sex is female and for age-class is adult.

|  | ***Elaphostrongylus alces*** | | | | | | | |
| --- | --- | --- | --- | --- | --- | --- | --- | --- |
|  | **Finnmark** | | | | **Trondelag** | | | |
|  | **b** | **SE** | **Chisq** | ***p*** | **b** | **SE** | **Chisq** | ***p*** |
| (Intercept) | **−** | **−** | **−** | **−** | -3.245 | 1.043 | 8.639 | **0.003** |
| Sex (male) | **−** | **−** | **−** | **−** | 0.822 | 0.805 | 0.952 | 0.329 |
| Age class (Calf) | **−** | **−** | **−** | **−** | 3.445 | 0.985 | 15.558 | **< 0.001** |
| Age class (Unknown) | **−** | **−** | **−** | **−** | 2.633 | 1.475 | 2.657 | 0.103 |
| Age class (Yearling) | **−** | **−** | **−** | **−** | 4.101 | 1.344 | 9.755 | **0.002** |
| prop. deciduous forest | **−** | **−** | **−** | **−** | **−** | **−** | **−** | **−** |
| prop. mixed forest | **−** | **−** | **−** | **−** | -5.207 | 12.521 | 0.153 | 0.696 |
| prop. agriculture | **−** | **−** | **−** | **−** | **−** | **−** | **−** | **−** |
| prop. marshland | **−** | **−** | **−** | **−** | -5.635 | 5.869 | 0.940 | 0.332 |
| prop. open land | **−** | **−** | **−** | **−** | 6.724 | 5.591 | 1.083 | 0.298 |
|  | **Gausdal-Murudal** | | | | **Valdres-Hallingdal** | | | |
|  | **b** | **SE** | **Chisq** | ***p*** | **b** | **SE** | **Chisq** | ***p*** |
| (Intercept) | -3.708 | 1.862 | 4.737 | **0.030** | -3.105 | 1.690 | 5.044 | **0.025** |
| Sex (male) | 1.789 | 0.989 | 3.295 | 0.069 | 1.268 | 1.373 | 0.496 | 0.481 |
| Age class (Calf) | **−** | **−** | **−** | **−** | **−** | **−** | **−** | **−** |
| Age class (Unknown) | **−** | **−** | **−** | **−** | 2.096 | 1.662 | 1.511 | 0.219 |
| Age class (Yearling) | 1.010 | 1.059 | 0.594 | 0.441 | 2.756 | 1.422 | 3.752 | 0.053 |
| prop. deciduous forest | -3.210 | 4.400 | 0.296 | 0.587 | 1.164 | 2.836 | 0.123 | 0.726 |
| prop. mixed forest | 1.723 | 6.359 | 0.048 | 0.826 | -0.187 | 5.716 | 0.001 | 0.979 |
| prop. agriculture | **−** | **−** | **−** | **−** | **−** | **−** | **−** | **−** |
| prop. marshland | 15.838 | 12.841 | 1.368 | 0.242 | -22.260 | 21.080 | 1.049 | 0.306 |
| prop. open land | 2.196 | 6.263 | 0.076 | 0.782 | 6.703 | 11.270 | 0.240 | 0.624 |
|  | **Hardangervidda** | | | |  |  |  |  |
|  | **b** | **SE** | **Chisq** | ***p*** |  |  |  |  |
| (Intercept) | -1.229 | 1.057 | 1.519 | 0.218 |  |  |  |  |
| Sex (male) | -0.010 | 1.357 | 0.000 | 0.994 |  |  |  |  |
| Age class (Calf) | -0.435 | 2.076 | 0.044 | 0.833 |  |  |  |  |
| Age class (Unknown) | 0.130 | 1.945 | 0.004 | 0.947 |  |  |  |  |
| Age class (Yearling) | **−** | **−** | **−** | **−** |  |  |  |  |
| prop. deciduous forest | -0.601 | 2.401 | 0.060 | 0.806 |  |  |  |  |
| prop. mixed forest | **−** | **−** | **−** | **−** |  |  |  |  |
| prop. agriculture | 0.173 | 2.442 | 0.005 | 0.944 |  |  |  |  |
| prop. marshland | **−** | **−** | **−** | **−** |  |  |  |  |
| prop. open land | 2.070 | 5.368 | 0.141 | 0.707 |  |  |  |  |

**Table S13 (cont.):** Results of Firth’s bias-reduced logistical regression testing the effect of habitat use patterns on the prevalence of six of the seven most common nematode taxa at each study area seperately. Habitat types constituting less than 3% proportional use in a study area were excluded from the model. The base value for the categorical variable sex is female and for age-class is adult.

|  | ***Trchostrongylus axei*** | | | | | | | |
| --- | --- | --- | --- | --- | --- | --- | --- | --- |
|  | **Finnmark** | | | | **Trondelag** | | | |
|  | **b** | **SE** | **Chisq** | ***p*** | **b** | **SE** | **Chisq** | ***p*** |
| (Intercept) | **−** | **−** | **−** | **−** | -1.734 | 0.824 | 2.987 | 0.084 |
| Sex (male) | **−** | **−** | **−** | **−** | 1.422 | 0.817 | 2.729 | 0.099 |
| Age class (Calf) | **−** | **−** | **−** | **−** | 0.154 | 0.884 | 0.027 | 0.870 |
| Age class (Unknown) | **−** | **−** | **−** | **−** | 0.367 | 1.607 | 0.044 | 0.834 |
| Age class (Yearling) | **−** | **−** | **−** | **−** | 2.377 | 1.260 | 2.990 | 0.084 |
| prop. deciduous forest | **−** | **−** | **−** | **−** | **−** | **−** | **−** | **−** |
| prop. mixed forest | **−** | **−** | **−** | **−** | -13.565 | 14.500 | 0.818 | 0.366 |
| prop. agriculture | **−** | **−** | **−** | **−** | **−** | **−** | **−** | **−** |
| prop. marshland | **−** | **−** | **−** | **−** | -0.712 | 6.489 | 0.011 | 0.917 |
| prop. open land | **−** | **−** | **−** | **−** | -8.166 | 8.027 | 0.822 | 0.365 |
|  | **Gausdal-Murudal** | | | | **Valdres-Hallingdal** | | | |
|  | **b** | **SE** | **Chisq** | ***p*** | **b** | **SE** | **Chisq** | ***p*** |
| (Intercept) | 0.854 | 1.116 | 0.522 | 0.470 | -1.168 | 0.988 | 1.399 | 0.237 |
| Sex (male) | 0.701 | 0.748 | 0.782 | 0.376 | -0.240 | 1.334 | 0.022 | 0.883 |
| Age class (Calf) | **−** | **−** | **−** | **−** | **−** | **−** | **−** | **−** |
| Age class (Unknown) | **−** | **−** | **−** | **−** | 0.408 | 1.232 | 0.099 | 0.753 |
| Age class (Yearling) | -1.574 | 1.476 | 1.212 | 0.271 | -0.308 | 1.662 | 0.025 | 0.875 |
| prop. deciduous forest | -8.810 | 4.699 | 3.721 | 0.054 | -3.696 | 4.172 | 0.563 | 0.453 |
| prop. mixed forest | -7.986 | 6.587 | 1.402 | 0.236 | -0.908 | 4.393 | 0.036 | 0.849 |
| prop. agriculture | **−** | **−** | **−** | **−** | **−** | **−** | **−** | **−** |
| prop. marshland | 2.750 | 9.606 | 0.068 | 0.794 | 11.802 | 22.098 | 0.167 | 0.683 |
| prop. open land | 2.522 | 6.971 | 0.085 | 0.771 | -7.065 | 9.750 | 0.385 | 0.535 |
|  | **Hardangervidda** | | | |  |  |  |  |
|  | **b** | **SE** | **Chisq** | ***p*** |  |  |  |  |
| (Intercept) | **−** | **−** | **−** | **−** |  |  |  |  |
| Sex (male) | **−** | **−** | **−** | **−** |  |  |  |  |
| Age class (Calf) | **−** | **−** | **−** | **−** |  |  |  |  |
| Age class (Unknown) | **−** | **−** | **−** | **−** |  |  |  |  |
| Age class (Yearling) | **−** | **−** | **−** | **−** |  |  |  |  |
| prop. deciduous forest | **−** | **−** | **−** | **−** |  |  |  |  |
| prop. mixed forest | **−** | **−** | **−** | **−** |  |  |  |  |
| prop. agriculture | **−** | **−** | **−** | **−** |  |  |  |  |
| prop. marshland | **−** | **−** | **−** | **−** |  |  |  |  |
| prop. open land | **−** | **−** | **−** | **−** |  |  |  |  |

**Table S13 (cont.):** Results of Firth’s bias-reduced logistical regression testing the effect of habitat use patterns on the prevalence of six of the seven most common nematode taxa at each study area seperately. Habitat types constituting less than 3% proportional use in a study area were excluded from the model. The base value for the categorical variable sex is female and for age-class is adult.

|  | ***Trchostrongylus spp.*** | | | | | | | |
| --- | --- | --- | --- | --- | --- | --- | --- | --- |
|  | **Finnmark** | | | | **Trondelag** | | | |
|  | **b** | **SE** | **Chisq** | ***p*** | **b** | **SE** | **Chisq** | ***p*** |
| (Intercept) | **−** | **−** | **−** | **−** | 0.125 | 0.702 | 0.027 | 0.870 |
| Sex (male) | **−** | **−** | **−** | **−** | -1.598 | 0.714 | 5.691 | **0.017** |
| Age class (Calf) | **−** | **−** | **−** | **−** | -0.488 | 0.788 | 0.378 | 0.539 |
| Age class (Unknown) | **−** | **−** | **−** | **−** | 0.772 | 1.327 | 0.316 | 0.574 |
| Age class (Yearling) | **−** | **−** | **−** | **−** | -0.251 | 1.092 | 0.053 | 0.818 |
| prop. deciduous forest | **−** | **−** | **−** | **−** | **−** | **−** | **−** | **−** |
| prop. mixed forest | **−** | **−** | **−** | **−** | -1.981 | 10.026 | 0.037 | 0.847 |
| prop. agriculture | **−** | **−** | **−** | **−** | **−** | **−** | **−** | **−** |
| prop. marshland | **−** | **−** | **−** | **−** | -3.462 | 7.287 | 0.186 | 0.666 |
| prop. open land | **−** | **−** | **−** | **−** | 0.932 | 4.010 | 0.052 | 0.820 |
|  | **Gausdal-Murudal** | | | | **Valdres-Hallingdal** | | | |
|  | **b** | **SE** | **Chisq** | ***p*** | **b** | **SE** | **Chisq** | ***p*** |
| (Intercept) | -0.015 | 1.103 | 0.000 | 0.990 | -3.224 | 1.584 | 6.644 | **0.010** |
| Sex (male) | 0.577 | 0.746 | 0.529 | 0.467 | 0.385 | 1.309 | 0.060 | 0.807 |
| Age class (Calf) | **−** | **−** | **−** | **−** | **−** | **−** | **−** | **−** |
| Age class (Unknown) | **−** | **−** | **−** | **−** | 1.873 | 1.534 | 1.406 | 0.236 |
| Age class (Yearling) | -0.480 | 1.325 | 0.118 | 0.732 | 0.902 | 1.449 | 0.315 | 0.575 |
| prop. deciduous forest | -0.973 | 2.830 | 0.098 | 0.754 | -0.302 | 2.934 | 0.008 | 0.928 |
| prop. mixed forest | -7.740 | 6.452 | 1.354 | 0.245 | 2.171 | 5.272 | 0.124 | 0.725 |
| prop. agriculture | **−** | **−** | **−** | **−** | **−** | **−** | **−** | **−** |
| prop. marshland | -13.207 | 11.539 | 1.237 | 0.266 | 3.516 | 18.956 | 0.027 | 0.868 |
| prop. open land | 3.147 | 5.782 | 0.231 | 0.631 | 7.307 | 9.287 | 0.536 | 0.464 |
|  | **Hardangervidda** | | | |  |  |  |  |
|  | **b** | **SE** | **Chisq** | ***p*** |  |  |  |  |
| (Intercept) | **−** | **−** | **−** | **−** |  |  |  |  |
| Sex (male) | **−** | **−** | **−** | **−** |  |  |  |  |
| Age class (Calf) | **−** | **−** | **−** | **−** |  |  |  |  |
| Age class (Unknown) | **−** | **−** | **−** | **−** |  |  |  |  |
| Age class (Yearling) | **−** | **−** | **−** | **−** |  |  |  |  |
| prop. deciduous forest | **−** | **−** | **−** | **−** |  |  |  |  |
| prop. mixed forest | **−** | **−** | **−** | **−** |  |  |  |  |
| prop. agriculture | **−** | **−** | **−** | **−** |  |  |  |  |
| prop. marshland | **−** | **−** | **−** | **−** |  |  |  |  |
| prop. open land | **−** | **−** | **−** | **−** |  |  |  |  |

**Table S13 (cont.):** Results of Firth’s bias-reduced logistical regression testing the effect of habitat use patterns on the prevalence of six of the seven most common nematode taxa at each study area seperately. Habitat types constituting less than 3% proportional use in a study area were excluded from the model. The base value for the categorical variable sex is female and for age-class is adult.

|  | **Unclassified 2 (Strongylida)** | | | | | | | |
| --- | --- | --- | --- | --- | --- | --- | --- | --- |
|  | **Finnmark** | | | | **Trondelag** | | | |
|  | **b** | **SE** | **Chisq** | ***p*** | **b** | **SE** | **Chisq** | ***p*** |
| (Intercept) | 2.218 | 1.969 | 1.529 | 0.216 | 1.507 | 0.676 | 5.622 | **0.018** |
| Sex (male) | -0.203 | 0.839 | 0.054 | 0.816 | 0.858 | 0.628 | 1.946 | 0.163 |
| Age class (Calf) | **−** | **−** | **−** | **−** | -1.159 | 0.700 | 2.827 | 0.093 |
| Age class (Unknown) | -0.732 | 1.087 | 0.378 | 0.539 | -1.232 | 1.245 | 0.940 | 0.332 |
| Age class (Yearling) | -0.385 | 1.834 | 0.042 | 0.839 | 0.326 | 1.101 | 0.088 | 0.767 |
| prop. deciduous forest | -0.590 | 2.208 | 0.056 | 0.813 | **−** | **−** | **−** | **−** |
| prop. mixed forest | **−** | **−** | **−** | **−** | -5.828 | 9.348 | 0.376 | 0.540 |
| prop. agriculture | **−** | **−** | **−** | **−** | **−** | **−** | **−** | **−** |
| prop. marshland | -0.009 | 6.095 | 0.000 | 0.999 | -7.990 | 6.085 | 1.934 | 0.164 |
| prop. open land | -1.577 | 4.349 | 0.107 | 0.744 | -3.950 | 4.019 | 0.951 | 0.329 |
|  | **Gausdal-Murudal** | | | | **Valdres-Hallingdal** | | | |
|  | **b** | **SE** | **Chisq** | ***p*** | **b** | **SE** | **Chisq** | ***p*** |
| (Intercept) | 0.351 | 1.022 | 0.113 | 0.737 | -1.167 | 0.910 | 1.764 | 0.184 |
| Sex (male) | -0.619 | 0.676 | 0.808 | 0.369 | 0.169 | 1.004 | 0.025 | 0.874 |
| Age class (Calf) | **−** | **−** | **−** | **−** | **−** | **−** | **−** | **−** |
| Age class (Unknown) | **−** | **−** | **−** | **−** | 3.409 | 1.594 | 7.466 | **0.006** |
| Age class (Yearling) | -1.724 | 1.048 | 2.869 | 0.090 | -0.787 | 1.344 | 0.311 | 0.577 |
| prop. deciduous forest | 1.151 | 2.347 | 0.238 | 0.625 | -0.473 | 2.306 | 0.039 | 0.844 |
| prop. mixed forest | 7.005 | 5.365 | 1.865 | 0.172 | -1.481 | 4.070 | 0.124 | 0.724 |
| prop. agriculture | **−** | **−** | **−** | **−** | **−** | **−** | **−** | **−** |
| prop. marshland | -2.925 | 8.538 | 0.113 | 0.737 | 14.320 | 16.069 | 0.745 | 0.388 |
| prop. open land | 0.433 | 4.855 | 0.008 | 0.931 | 0.623 | 6.390 | 0.009 | 0.924 |
|  | **Hardangervidda** | | | |  |  |  |  |
|  | **b** | **SE** | **Chisq** | ***p*** |  |  |  |  |
| (Intercept) | -1.229 | 1.057 | 1.519 | 0.218 |  |  |  |  |
| Sex (male) | -0.010 | 1.357 | 0.000 | 0.994 |  |  |  |  |
| Age class (Calf) | -0.435 | 2.076 | 0.044 | 0.833 |  |  |  |  |
| Age class (Unknown) | 0.130 | 1.945 | 0.004 | 0.947 |  |  |  |  |
| Age class (Yearling) | **−** | **−** | **−** | **−** |  |  |  |  |
| prop. deciduous forest | -0.601 | 2.401 | 0.060 | 0.806 |  |  |  |  |
| prop. mixed forest | **−** | **−** | **−** | **−** |  |  |  |  |
| prop. agriculture | 0.173 | 2.442 | 0.005 | 0.944 |  |  |  |  |
| prop. marshland | **−** | **−** | **−** | **−** |  |  |  |  |
| prop. open land | 2.070 | 5.368 | 0.141 | 0.707 |  |  |  |  |

**Table S13 (cont.):** Results of Firth’s bias-reduced logistical regression testing the effect of habitat use patterns on the prevalence of six of the seven most common nematode taxa at each study area seperately. Habitat types constituting less than 3% proportional use in a study area were excluded from the model. The base value for the categorical variable sex is female and for age-class is adult.

**Migration and nematode taxa richness (global model)**

|  | **b** | **SE** | **t-value** | ***p*** |
| --- | --- | --- | --- | --- |
| (Intercept) | 3.044 | 0.894 | 3.405 | **0.001** |
| Sex (male) | 0.174 | 0.178 | 0.975 | 0.331 |
| Age Class (Calf) | 1.165 | 0.393 | 2.964 | **0.004** |
| Age Class (Unknown) | 0.398 | 0.362 | 1.099 | 0.274 |
| Age Class (Yearling) | 0.716 | 0.317 | 2.256 | **0.025** |
| Study Area (Gausdal-Murudal) | -8.847 | 2.083 | -4.247 | **< 0.001** |
| Study Area (Hardangervidda) | -0.003 | 2.342 | -0.001 | 0.999 |
| Study Area (Trøndelag) | -3.392 | 1.441 | -2.353 | **0.020** |
| Study Area (Valdres-Hallingdal) | -0.281 | 1.694 | -0.166 | 0.868 |
| log (migration distance) | 0.029 | 0.093 | 0.307 | 0.759 |
| Elevation Change | -0.004 | 0.002 | -1.809 | 0.072 |
| Study Area (Gausdal-Murudal):log(migration distance) | 0.848 | 0.205 | 4.132 | **< 0.001** |
| Study Area (Hardangervidda):log(miggration distance) | -0.099 | 0.260 | -0.381 | 0.704 |
| Study Area (Trøndelag:log(migration distance) | 0.389 | 0.169 | 2.295 | **0.023** |
| Study Area (Valdres-Hallingdal):log(migration distance) | -0.043 | 0.199 | -0.217 | 0.828 |
| Study Area (Gausdal-Murudal):Elevation Change | 0.006 | 0.002 | 2.502 | **0.013** |
| Study Area (Hardangervidda):Elevation Change | 0.006 | 0.004 | 1.613 | 0.109 |
| Study Area (Trøndelag):Elevation Change | 0.000 | 0.002 | 0.175 | 0.862 |
| Study Area (Valdres-Hallingdal):Elevation Change | 0.003 | 0.002 | 1.362 | 0.175 |

**Table S14:** LMM results for the global migration model testing the effect of individual migration parameters on nematode taxa richness across all study areas. The base value for the categorical variable sex is female, for age-class is adult and study area is Finnmark.

**Migration and nematode beta-diversity (global model)**

|  | **SS** | **R^2^** | **F** | ***p*** |
| --- | --- | --- | --- | --- |
| Sex | 0.427 | 0.013 | 2.786 | **0.014** |
| Age Class | 1.371 | 0.041 | 2.978 | **0.001** |
| Year | 0.311 | 0.009 | 2.025 | 0.068 |
| Study Area | 3.153 | 0.095 | 5.138 | **0.001** |
| Elevation Change | 0.227 | 0.007 | 1.479 | 0.158 |
| Migration Distance | 0.612 | 0.018 | 3.989 | **0.003** |
| Study Area:Migration Distance | 0.721 | 0.022 | 1.175 | 0.274 |
| Study Area:Elevation Change | 0.549 | 0.017 | 0.895 | 0.598 |

**Table S15:** PERMANOVA results testing the effect of individual migration parameters on nematode taxa beta-diversity across all study areas based on Jaccard dissimilarity.

**Migration and nematode taxa prevalence (global model)**

|  | ***Nematodirella* spp.** | | | | ***Elaphostrongylus alces*** | | | | ***Trichostrongylus axei*** | | | |
| --- | --- | --- | --- | --- | --- | --- | --- | --- | --- | --- | --- | --- |
|  | **coef** | **SE** | **Chisq** | ***p*** | **coef** | **SE** | **Chisq** | ***p*** | **coef** | **SE** | **Chisq** | ***p*** |
| (Intercept) | -0.132 | 1.281 | 0.011 | 0.918 | -1.463 | 1.591 | 1.011 | 0.315 | -1.544 | 1.698 | 1.017 | 0.313 |
| Sex (Male) | -0.239 | 0.340 | 0.482 | 0.487 | 1.036 | 0.508 | 3.663 | 0.056 | 0.219 | 0.472 | 0.175 | 0.675 |
| Age Class (calf) | 1.297 | 0.694 | 3.793 | 0.051 | 2.588 | 0.752 | 12.039 | **0.001** | 0.289 | 0.839 | 0.104 | 0.747 |
| Age Class (unknown) | 0.402 | 0.550 | 0.536 | 0.464 | 2.202 | 0.906 | 4.069 | **0.044** | 0.166 | 0.864 | 0.029 | 0.865 |
| Age Class (yearling) | 0.614 | 0.589 | 1.107 | 0.293 | 2.171 | 0.691 | 8.705 | **0.003** | 0.609 | 0.899 | 0.339 | 0.560 |
| Study Area (Gausdal-Murudal) | -10.137 | 4.364 | 6.265 | **0.012** | 2.205 | 4.799 | 0.177 | 0.674 | -26.643 | 9.984 | 7.283 | **0.007** |
| Study Area (Hardangervidda) | -4.720 | 5.708 | 0.633 | 0.426 | 8.403 | 5.924 | 1.867 | 0.172 | -2.967 | 7.530 | 0.118 | 0.731 |
| Study Area Trøndelag) | -0.445 | 2.501 | 0.031 | 0.859 | -3.272 | 3.750 | 0.686 | 0.407 | -1.471 | 3.504 | 0.154 | 0.694 |
| Study Area (Valdres-Hallingdal) | -0.237 | 2.771 | 0.007 | 0.932 | -11.392 | 6.971 | 3.177 | 0.075 | 2.720 | 4.010 | 0.376 | 0.540 |
| log (migration distance) | 0.041 | 0.146 | 0.078 | 0.780 | -0.467 | 0.227 | 2.091 | 0.148 | -0.033 | 0.194 | 0.023 | 0.881 |
| Elevation Change | -0.002 | 0.004 | 0.387 | 0.534 | 0.001 | 0.002 | 0.169 | 0.681 | -0.006 | 0.005 | 1.141 | 0.285 |
| Study Area (Gausdal-Murudal):log(migration distance) | 1.055 | 0.438 | 6.709 | **0.010** | 0.137 | 0.505 | 0.066 | 0.798 | 2.563 | 0.921 | 7.131 | **0.008** |
| Study Area (Hardangervidda):log(migration distance) | 0.728 | 0.693 | 1.108 | 0.293 | -0.679 | 0.727 | 0.803 | 0.370 | 0.246 | 0.831 | 0.064 | 0.800 |
| Study Area (Trøndelag):log(migration distance) | 0.024 | 0.300 | 0.007 | 0.935 | 0.670 | 0.454 | 1.937 | 0.164 | 0.195 | 0.416 | 0.191 | 0.662 |
| Study Area (Valdres-Hallingdal):log(migration distance) | -0.010 | 0.345 | 0.001 | 0.977 | 1.443 | 0.731 | 4.360 | **0.037** | -0.395 | 0.531 | 0.458 | 0.498 |
| Study Area (Gausdal-Murudal):Elevation Change | 0.009 | 0.005 | 3.806 | 0.051 | **−** | **−** | **−** | **−** | 0.007 | 0.006 | 1.124 | 0.289 |
| Study Area (Hardangervidda):Elevation Change | 0.005 | 0.008 | 0.283 | 0.595 | **−** | **−** | **−** | **−** | 0.004 | 0.010 | 0.093 | 0.760 |
| Study Area (Trøndelag):Elevation Change | 0.002 | 0.004 | 0.151 | 0.698 | **−** | **−** | **−** | **−** | 0.000 | 0.006 | 0.004 | 0.949 |
| Study Area (Valdres-Hallingdal):Elevation Change | 0.003 | 0.004 | 0.466 | 0.495 | **−** | **−** | **−** | **−** | 0.007 | 0.007 | 0.846 | 0.358 |

**Table 16:** Firth’s bias-reduced logistic regression testing the relationship between migration parameters and prevalence of the most common nematodes across all study areas. The base value for the categorical variable sex is female, for age-class is adult and study area is Finnmark.

**Migration and nematode taxa prevalence (global model)(cont.)**

|  | ***Trichostrongylus* spp.** | | | | **Unclassified Strongylida** | | | |
| --- | --- | --- | --- | --- | --- | --- | --- | --- |
|  | **coef** | **SE** | **Chisq** | ***p*** | **coef** | **SE** | **Chisq** | ***p*** |
| (Intercept) | -2.095 | 2.048 | 1.471 | 0.225 | 3.004 | 1.525 | 4.461 | **0.035** |
| Sex (Male) | -0.576 | 0.448 | 1.517 | 0.218 | 0.106 | 0.362 | 0.082 | 0.775 |
| Age Class (calf) | -0.827 | 0.778 | 1.171 | 0.279 | -0.804 | 0.627 | 1.644 | 0.200 |
| Age Class (unknown) | 0.644 | 0.739 | 0.610 | 0.435 | 0.677 | 0.657 | 1.041 | 0.308 |
| Age Class (yearling) | 0.155 | 0.771 | 0.036 | 0.850 | -0.782 | 0.597 | 1.704 | 0.192 |
| Study Area (Gausdal-Murudal) | -6.107 | 6.201 | 0.618 | 0.432 | -0.319 | 0.727 | 0.187 | 0.665 |
| Study Area (Hardangervidda) | -3.121 | 7.627 | 0.128 | 0.721 | -3.880 | 1.076 | 22.029 | **< 0.001** |
| Study Area Trøndelag) | -1.918 | 3.194 | 0.211 | 0.646 | -1.118 | 0.685 | 2.865 | 0.091 |
| Study Area (Valdres-Hallingdal) | 3.790 | 4.191 | 0.698 | 0.404 | -2.069 | 0.818 | 6.824 | **0.009** |
| log (migration distance) | 0.050 | 0.229 | 0.040 | 0.842 | -0.159 | 0.157 | 0.965 | 0.326 |
| Elevation Change | -0.009 | 0.006 | 2.661 | 0.103 | -0.001 | 0.005 | 0.051 | 0.821 |
| Study Area (Gausdal-Murudal):log(migration distance) | 0.596 | 0.595 | 0.580 | 0.446 | **−** | **−** | **−** | **−** |
| Study Area (Hardangervidda):log(migration distance) | 0.280 | 0.844 | 0.081 | 0.776 | **−** | **−** | **−** | **−** |
| Study Area (Trøndelag):log(migration distance) | 0.396 | 0.373 | 0.435 | 0.510 | **−** | **−** | **−** | **−** |
| Study Area (Valdres-Hallingdal):log(migration distance) | -0.622 | 0.563 | 1.085 | 0.298 | **−** | **−** | **−** | **−** |
| Study Area (Gausdal-Murudal):Elevation Change | 0.008 | 0.006 | 1.458 | 0.227 | 0.006 | 0.006 | 1.036 | 0.309 |
| Study Area (Hardangervidda):Elevation Change | 0.008 | 0.010 | 0.572 | 0.450 | 0.015 | 0.011 | 2.038 | 0.153 |
| Study Area (Trøndelag):Elevation Change | 0.007 | 0.006 | 1.335 | 0.248 | 0.002 | 0.005 | 0.131 | 0.718 |
| Study Area (Valdres-Hallingdal):Elevation Change | 0.015 | 0.007 | 4.154 | **0.042** | 0.001 | 0.005 | 0.021 | 0.885 |

**Table 16 (cont.):** Firth’s bias-reduced logistic regression testing the relationship between migration parameters and prevalence of the most common nematodes across all study areas. The base value for the categorical variable sex is female, for age-class is adult, and study area is Finnmark.


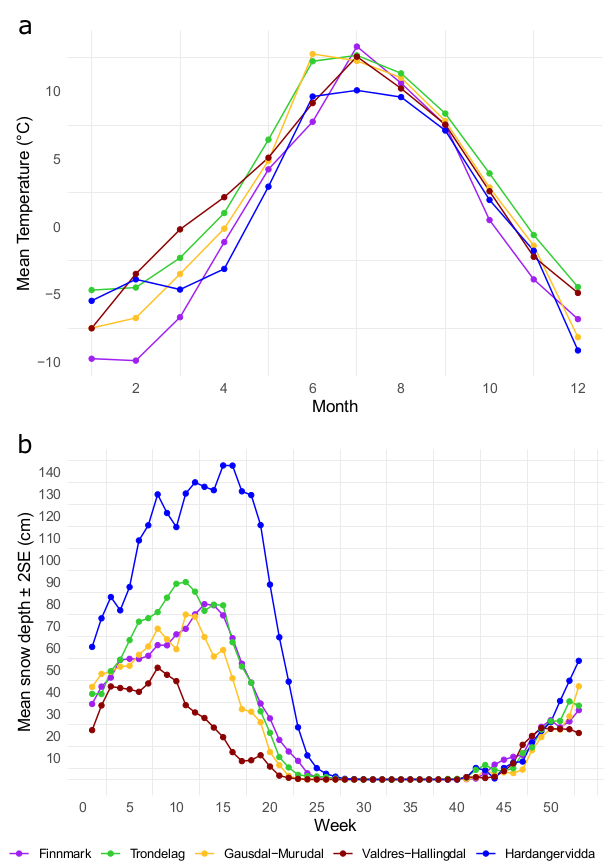


**Fig. S1:** Mean monthly temperatures (a) and weekly snow depth (b) at moose GPS positions in each of the five study areas. Estimates from 1 × 1 km gridded data based on observational data and interpolation methods (Lussana et al., 2019; Saloranta, 2016).


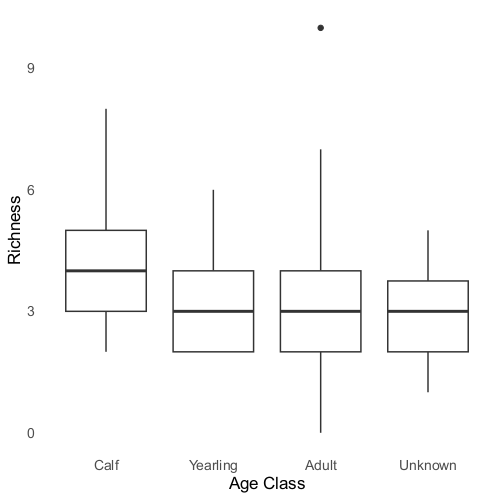


**Fig. S2:** Nematode richness among age classes.

**
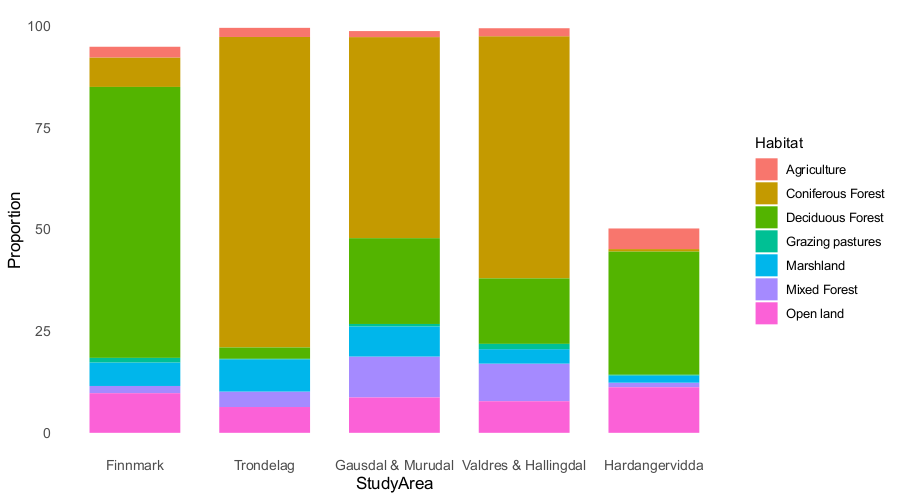
**

**Fig. S3:** Average proportional use of each habitat type by moose at each study area based on GPS data. Land cover types of fresh water, sea, glaciers, and built-up areas are excluded.
